# Machine learning-based prediction of in-ICU mortality in pneumonia patients

**DOI:** 10.1038/s41598-023-38765-8

**Published:** 2023-07-17

**Authors:** Eun-Tae Jeon, Hyo Jin Lee, Tae Yun Park, Kwang Nam Jin, Borim Ryu, Hyun Woo Lee, Dong Hyun Kim

**Affiliations:** 1 Department of Radiology, Seoul National University College of Medicine, Seoul Metropolitan Government-Seoul National University Boramae Medical Center, 5 Gil 20, Boramae-Road, Dongjak-gu, Seoul, South Korea; 2 Division of Respiratory and Critical Care, Department of Internal Medicine, Seoul National University College of Medicine, Seoul Metropolitan Government-Seoul National University Boramae Medical Center, 5 Gil 20, Boramae-Road, Dongjak-gu, Seoul, South Korea; 3 Center for Data Science, Biomedical Research Institute, Seoul Metropolitan Government-Seoul National University Boramae Medical Center, Seoul, South Korea

**Keywords:** Diseases, Medical research

## Abstract

Conventional severity-of-illness scoring systems have shown suboptimal performance for predicting in-intensive care unit (ICU) mortality in patients with severe pneumonia. This study aimed to develop and validate machine learning (ML) models for mortality prediction in patients with severe pneumonia. This retrospective study evaluated patients admitted to the ICU for severe pneumonia between January 2016 and December 2021. The predictive performance was analyzed by comparing the area under the receiver operating characteristic curve (AU-ROC) of ML models to that of conventional severity-of-illness scoring systems. Three ML models were evaluated: (1) logistic regression with L2 regularization, (2) gradient-boosted decision tree (LightGBM), and (3) multilayer perceptron (MLP). Among the 816 pneumonia patients included, 223 (27.3%) patients died. All ML models significantly outperformed the Simplified Acute Physiology Score II (AU-ROC: 0.650 [0.584–0.716] vs 0.820 [0.771–0.869] for logistic regression vs 0.827 [0.777–0.876] for LightGBM 0.838 [0.791–0.884] for MLP; P < 0.001). In the analysis for NRI, the LightGBM and MLP models showed superior reclassification compared with the logistic regression model in predicting in-ICU mortality in all length of stay in the ICU subgroups; all age subgroups; all subgroups with any APACHE II score, PaO_2_/FiO_2_ ratio < 200; all subgroups with or without history of respiratory disease; with or without history of CVA or dementia; treatment with mechanical ventilation, and use of inotropic agents. In conclusion, the ML models have excellent performance in predicting in-ICU mortality in patients with severe pneumonia. Moreover, this study highlights the potential advantages of selecting individual ML models for predicting in-ICU mortality in different subgroups.

## Introduction

Pneumonia is among the leading causes of death globally^1^, and each year, 0.1 million patients with pneumonia require intensive care unit (ICU) admission for mechanical ventilation (MV)^2,3^. Approximately half of the patients with respiratory failure in the ICU have pneumonia, and a quarter of them are re-admitted to the ICU^4^. Approximately 20–50% of patients with pneumonia who are admitted to the ICU die^4–7^. Clinical scoring systems to predict in-ICU mortality in critically ill patients have been introduced, but none of these have been specifically developed for patients admitted to the ICU with severe pneumonia. Therefore, there is an urgent need for severity assessment tools for predicting in-ICU mortality in patients with severe pneumonia^8^.

There has been recent interest in identifying the factors related to the outcomes of ICU patients. Vital signs^9–11^; laboratory markers such as lactate, thrombocytopenia, troponin, and bicarbonate levels^12–14^; and intervention strategies such as the use of vasopressors, MV, and continuous renal replacement therapy have been identified to be closely related to the prognoses of ICU patients^15,16^. However, previously validated predictive models including CURB-65, CRB-65, Pneumonia Severity Index (PSI), Quick Sequential Organ Failure Assessment (qSOFA), Simplified Acute Physiology Score 3 (SAPS-3), and Acute Physiology and Chronic Health Evaluation II (APACHE II) had suboptimal predictive performance in patients with severe pneumonia at ICU admission or emergency room department visits^17–19^. Therefore, a new prediction model for in-ICU mortality of patients admitted to the ICU for severe pneumonia needs to be developed.

Machine learning (ML) has demonstrated great potential in various medical fields, including surgery^20^, cardiology^21,22^ (early detection of heart failure)^23^, cancer research^24,25^, and intensive care medicine (diagnosis or prognosis prediction of critical illness)^26–28^. Although the integration of ML into the ICU setting is still in its early stages, several studies have already explored its application in managing critically ill patients^26^. Some studies have used large population datasets to predict length of stay (LOS)^29^, ICU readmission^30^, mortality rates^31–33^, and the risks of sepsis^34^ or acute respiratory distress syndrome^35^. Particular, there is increasing evidence that ML models are superior to conventional severity-of-illness scoring systems for predicting mortality in ICU patients^36,37^ or patients with pneumonia^38^. In addition, ML models may have good predictive performance for mortality, especially in patients with severe pneumonia^39–41^. One study demonstrated that the artificial neural network model had strong predictive performance for 14-day hospital readmission in patients with pneumonia^42^. Another study demonstrated that ML model has good performance for predicting 30-day mortality in sepsis patients^28^. However, evidence on whether ML models can accurately predict in-ICU mortality in patients with severe pneumonia who require ICU admission is rare.

Thus, the present study aimed to elucidate the performance of ML models in predicting mortality in patients with severe pneumonia who require ICU admission, using the information at the time of ICU admission. Our findings will aid in selecting individual ML models for predicting in-ICU mortality in different subgroups.

## Methods

### Ethics

This study complied with the Transparent Reporting of a multivariable prediction model for Individual Prognosis or Diagnosis (TRIPOD) reporting guidelines^43^. This study was approved by the IRB Committee of Seoul National University Seoul Metropolitan Government (SNU-SMG) Boramae Medical Center (IRB Number: 10-2021-110) on November 16, 2022 and was conducted in accordance with the 1975 ethical guidelines of the Declaration of Helsinki. The requirement for informed consent from the study subjects was waived by the IRB Committee of Seoul National University Seoul Metropolitan Government (SNU-SMG) Boramae Medical Center due to the retrospective study design.

### Data collection, study design, and population

This observational study retrospectively evaluated patients admitted to the ICU for pneumonia at the SNU-SMG Boramae Medical Center between January 2016 and December 2021. The inclusion criteria were: (1) age ≥ 18 years; (2) ICU admission; (3) International Classification of Disease 10th edition code for pneumonia as a major diagnosis or detection of pneumonia on chest computed tomography within 1 week of ICU admission; (4) C-reactive protein (CRP) level ≥ 4 mg/dL; and (5) use of antibiotics for pneumonia. The exclusion criteria were: (1) no oxygen requirement; (2) transfer to the general ward within 3 days; and (3) ICU admission due to more serious medical conditions other than pneumonia.

Baseline data including age, sex, body mass index, smoking history, previous underlying disease, and respiratory comorbidities and clinical features were collected. Clinical features included prognostic scores, vital signs, laboratory examination results, and treatment with antibiotics or steroids.

### Main outcome measures

The primary outcome measure was the prognostic accuracy of ML models compared with that of conventional severity-of-illness scoring systems for predicting in-ICU mortality in patients who required ICU admission for severe pneumonia. The secondary outcome measures were as follows: (1) the prognostic accuracy of the ML models; (2) the prognostic accuracy of the ML models in different subgroups; (3) the clinical factors contributing to the prediction of in-ICU mortality in patients admitted to the ICU for severe pneumonia. ICU admission due to severe pneumonia was determined as the presence of at least one major criterion or three minor criteria of the Infectious Disease Society of America/American Thoracic Society guidelines^3^.

The conventional severity-of-illness scoring systems included the SOFA, SAPS II, and APACHE II scores. Among the scoring systems, the best model that showed the strongest performance was used as the baseline comparator. For the statistical and ML models, we tested three popular models: (1) logistic regression with L2 regularization, (2) gradient-boosted decision tree (LightGBM), and (3) multilayer perceptron (MLP).

### Data splitting and preprocessing

Variables with more than a 20% missing rate were excluded to generate the available dataset. Approximately 40% of the data were randomly separated with stratification by the outcome and subgrouping variables. The held-out data were used as a test set only for internal validation of the models. The remaining data were used to develop the models as a training set in a tenfold cross-validation scheme.

Missing values were imputed using multivariate imputation by chained equations^44^. Outliers were detected using an isolation forest^45^ and subsequently replaced with the closest normal value of the training set. All the variables included in the analysis and their missing rates are listed in Supplementary Table S1.

### Variable importance and feature selection

The influence of each variable on the predictive ability of the model was evaluated using the SHapley Additive exPlanations (SHAP) method^46^. To rank the variables, the mean absolute SHAP values were calculated as the relative importance of the variables. A LightGBM was used for the SHAP evaluation^47^. The guiding metric for cross-validation performance was the area under the receiver operating characteristic curve (AU-ROC).

### Model development

Supplementary Fig. S1 presents the workflow diagram for model development. LightGBM is a gradient-boosted tree-based ensemble model, whereas MLP is a feedforward neural network with a basic architecture comprising fully connected layers. The hyperparameters were tuned using Bayesian optimization to maximize the cross-validation performance. Details of the hyperparameter tuning with package information for all tested models are described in Supplementary Methods and Supplementary Table S2. The models were calibrated using isotonic regression according to the validation data obtained during cross-validation. The methods in the present study were implemented in Python version 3.9.7 (Python Software Foundation, Wilmington, Del, USA), with scikit-learn (version 1.1.2).

### Internal validation in different subgroups

The model performance in the different patient subgroups was evaluated using the test set. We prespecified subgroups based on the clinically important phenotypes of pneumonia in the ICU^48^. Subgroup analyses were performed according to (1) the period from hospital admission to ICU admission, (2) age, (3) APACHE II scores, (4) PaO_2_/FiO_2_ ratio, (5) history of chronic respiratory disease, (6) history of cerebrovascular accident (CVA) or dementia, (7) MV, and (8) use of vasopressors.

### Statistical analysis

Categorical variables were analyzed using the chi-squared test, while continuous variables were analyzed using the independent *t*-test or Mann–Whitney U test. We evaluated the AU-ROC as an overall performance measure and compared the models using the Delong method^49^. Owing to an imbalance in outcome prevalence, the area under the precision-recall curve (AU-PRC) was also evaluated as another overall performance measure^50^. Furthermore, the performance of the models was evaluated in detail according to sensitivity, positive predictive value, negative predictive value, diagnostic odds ratio, and net reclassification improvement (NRI)^51^ at three low false-positive rates (FPR) levels of 10%, 20%, and 30%. The recalibration effects were also evaluated using decision curves, which presented a net benefit against different decision thresholds^52^. The sensitivity at fixed FPR levels was evaluated in the subgroups using the best-performing model for overall performance. Calibration errors were evaluated before and after calibration using the Brier score and calibration curves. The Brier score was calculated as the mean squared error of the predicted probabilities^53^. *P* values were adjusted using the Benjamini–Hochberg method for multiple comparisons, and the significance level was set at *P* < 0.05.

## Results

### Baseline characteristics and clinical features

**Figure 1:**
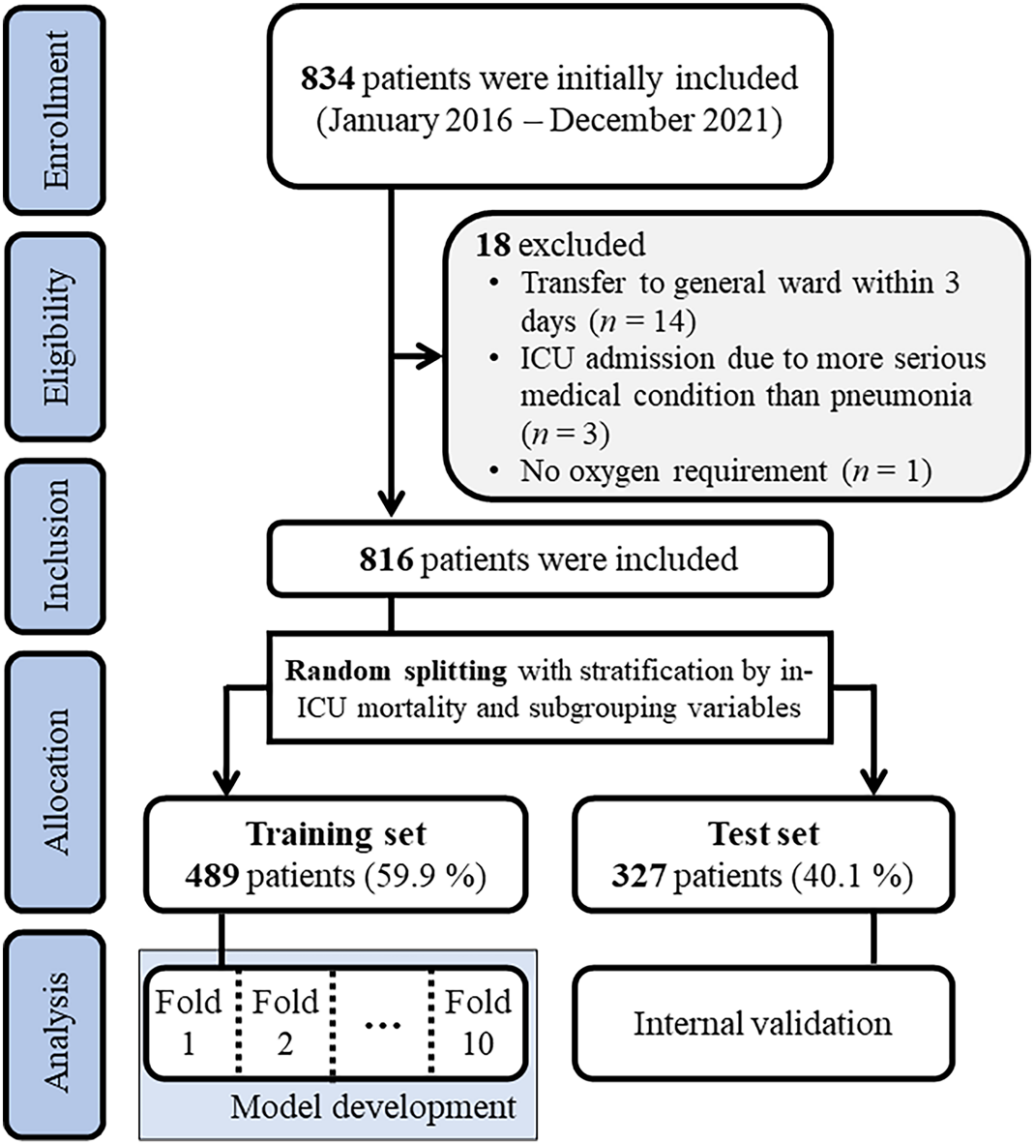
Patient selection flowchart.

In total, 816 patients with pneumonia admitted to the ICU were included in the analysis (Fig. 1). The median patient age was 77 years, and 588 patients (72.1%) were male. Overall, 223 (27.3%) patients died. The median duration of LOS was 6 days (IQR, 2–13 days). The baseline characteristics are summarized in Table 1. There were significant differences in age and smoking history between the survivor and non-survivor groups. The non-survivor group was more likely to be older, involve a higher number of current smokers, and had a higher number of smoking pack-years among ever-smokers (9.9). Regarding comorbidities, the non-survivor group also included more patients with interstitial lung disease, pulmonary tuberculosis, lung cancer, chronic kidney disease, cerebellar vessel disease, cardiovascular disease, chronic heart failure, and metastatic cancer. Further, this group had higher illness severity scores, including the APACHE II, SOFA, and SAPS II scores.

**Table 1:** Baseline patient characteristics.

Variable	All	Survivors	Non-survivors	*P* value
	(n = 816)	(n = 593, 72.7%)	(n = 223, 27.3%)	
Demographics				
Age (yrs), median (IQR)	77 (67–83)	76 (66–83)	78 (68–85)	0.050
Males, n (%)	588 (72.1%)	427 (72.0%)	161 (72.2%)	1.000
BMI (kg/m^2^), mean (SD)	20.7 (4.5)	20.8 (4.6)	20.3 (4.1)	0.255
Smoking				0.002
Ex-smoker, n (%)	233 (28.6%)	173 (29.2%)	60 (26.9%)	
Current smoker, n (%)	43 (5.3%)	21 (3.5%)	22 (9.9%)	
Pack years in ever-smokers, mean (SD)	9.9 (19.0)	9.1 (18.0)	11.8 (21.1)	0.083
Underlying comorbidities				
Hypertension, n (%)	450 (55.1%)	334 (56.3%)	116 (52.0%)	0.306
Diabetes, n (%)	319 (39.1%)	240 (40.5%)	79 (35.4%)	0.216
COPD, n (%)	62 (7.6%)	42 (7.1%)	20 (9.0%)	0.449
Asthma, n (%)	24 (2.9%)	16 (2.7%)	8 (3.6%)	0.662
Interstitial lung disease, n (%)	31 (3.8%)	14 (2.4%)	17 (7.6%)	< 0.001
Nontuberculous mycobacteria, n (%)	8 (1.0%)	4 (0.7%)	4 (1.8%)	0.295
Tuberculosis, n (%)	119 (14.6%)	82 (13.8%)	37 (16.6%)	0.376
Lung cancer, n (%)	11 (1.3%)	4 (0.7%)	7 (3.1%)	0.017
Chronic kidney disease, n (%)	121 (14.8%)	77 (13.0%)	44 (19.7%)	0.021
Chronic liver disease, n (%)	48 (5.9%)	34 (5.7%)	14 (6.3%)	0.898
Cardiovascular disease, n (%)	14 (1.7%)	5 (0.8%)	9 (4.0%)	0.005
Congestive heart failure, n (%)	32 (3.9%)	14 (2.4%)	18 (8.1%)	< 0.001
Cerebrovascular accident, n (%)	11 (1.3%)	5 (0.8%)	6 (2.7%)	0.089
Dementia, n (%)	63 (7.7%)	44 (7.4%)	19 (8.5%)	0.706
HIV, n (%)	5 (0.6%)	3 (0.5%)	2 (0.9%)	0.893
Metastatic cancer, n (%)	11 (1.3%)	3 (0.5%)	8 (3.6%)	0.002
Hematologic malignancy, n (%)	2 (0.2%)	1 (0.2%)	1 (0.4%)	1.000

BMI, body mass index; COPD, chronic obstructive pulmonary disease; HIV, human immunodeficiency virus.

The clinical characteristics are presented in Table 2. Regarding vital signs, the survivor group had higher systolic, diastolic, or mean blood pressure, while the non-survivor group had faster heart and respiratory rates and lower urine output. The non-survivor group had lower levels of partial pressure of oxygen (PaO_2_) or carbon dioxide (HCO_3−_), lower levels of oxygen saturation (SpO_2_), and a lower ratio of PaO_2_/FiO_2_. For laboratory findings, the levels of urea nitrogen, creatinine, alanine aminotransferase, total bilirubin, and alkaline phosphatase (ALP) and the prothrombin time and international normalized ratio (PT-INR) were higher in the non-survivor group. Meanwhile, the survivor group was more likely to be treated with steroids and vasopressors. The comparison results between the training and test sets for the baseline characteristics and clinical features are presented in Supplementary Tables S3 and S4, respectively.

**Table 2:** Clinical patient characteristics.

Variable	All	Survivors	Non-survivors	*P* value
	(n = 816)	(n = 593, 72.7%)	(n = 223, 27.3%)	
Prognostic scores				
APACHE II, median (IQR)	21 (16–28)	20 (15–26)	25 (19–31)	< 0.001
SOFA, median (IQR)	10 (7–13)	9 (7–12)	12 (8–14)	< 0.001
SAPS II, median (IQR)	48 (37–62)	45 (35–58)	56 (45–71)	< 0.001
Initial vital signs				
Glasgow Coma Scale, median (IQR)	11 (6–14)	11 (6–14)	10 (6–14)	0.523
Initial SBP (mmHg), median (IQR)	122.0 (102.0–146.0)	126.0 (108.0–151.0)	110.0 (91.0–132.0)	< 0.001
Initial DBP (mmHg), median (IQR)	64.0 (54.0–75.2)	65.0 (54.0–77.0)	61.0 (50.0–71.5)	< 0.001
Initial MBP, median (IQR)	84.3 (70.7–98.0)	86.7 (73.3–100.7)	78.7 (62.8–89.3)	< 0.001
Initial PR (beats/minute), median (IQR)	104.0 (86.0–120.0)	102.0 (84.0–117.0)	112.0 (94.0–130.0)	< 0.001
Initial RR (breaths/minute), median (IQR)	22.0 (19.0–27.0)	22.0 (18.0–26.0)	24.0 (20.0–28.0)	< 0.001
Body temperature (°C), mean (SD)	36.9 (1.1)	36.8 (1.1)	37.0 (1.2)	0.100
Urine output (ml/hour), mean (SD)	66.0 (52.6)	73.0 (55.1)	47.3 (39.5)	< 0.001
Laboratory findings				
pH, median (IQR)	7.4 (7.3–7.5)	7.4 (7.3–7.4)	7.4 (7.2–7.5)	0.095
pCO_2_ (mmHg), median (IQR)	35.3 (29.2–43.7)	35.9 (29.6–43.3)	33.4 (27.6–45.3)	0.122
PaO_2_ (mmHg), median (IQR)	68.3 (54.0–85.7)	70.7 (55.6–88.5)	63.2 (51.5–76.5)	< 0.001
HCO_3_- (mEq/L), median (IQR)	21.0 (17.1–24.7)	21.7 (17.9–25.3)	19.6 (15.4–22.9)	< 0.001
Initial SpO_2_, median (IQR)	95.0 (90.0–98.0)	96.0 (92.0–98.0)	93.0 (87.2–97.0)	< 0.001
PaO_2_/FiO_2_ ratio, median (IQR)	121.6 (78.3–194.0)	139.7 (88.6–212.4)	85.7 (63.3–127.0)	< 0.001
White blood cell (10^3^/μL), median (IQR)	11.2 (7.6–16.3)	11.1 (7.6–15.5)	12.0 (7.5–18.7)	0.148
Neutrophil (10^3^/μL), median (IQR)	84.0 (75.9–89.2)	84.0 (76.0–89.4)	84.1 (75.5–88.8)	0.665
Lymphocyte (10^3^/μL), median (IQR)	10.1 (6.6–16.0)	10.2 (6.9–15.8)	9.9 (6.0–16.2)	0.274
Hemoglobin, median (IQR)	10.9 (9.2–12.5)	11.1 (9.4–12.6)	10.6 (8.9–11.9)	0.003
Platelet (10^3^/μL), median (IQR)	205.0 (134.0–294.2)	209.0 (149.0–301.0)	187.0 (96.0–279.5)	0.003
Lactate (mmol/L), mean (SD)	4.3 (5.2)	3.6 (4.1)	6.1 (6.8)	< 0.001
C-reactive protein (mg/L), median (IQR)	13.4 (5.8–22.0)	11.8 (4.8–20.8)	17.2 (10.2–24.0)	< 0.001
Procalcitonin (ng/mL), mean (SD)	2.4 (7.7)	2.3 (8.4)	2.7 (5.2)	0.706
BUN (mg/dL), median (IQR)	27.0 (15.0–44.0)	26.0 (15.0–42.0)	29.0 (17.0–51.5)	0.031
Creatinine (mg/dL), median (IQR)	1.2 (0.8–2.1)	1.1 (0.8–1.9)	1.4 (0.9–2.3)	0.006
Na (mEq/L), median (IQR)	136.0 (132.2–139.8)	136.2 (132.8–139.9)	135.1 (131.2–139.1)	0.027
K (mEq/L), median (IQR)	4.1 (3.6–4.7)	4.1 (3.6–4.6)	4.1 (3.5–4.7)	0.986
TCO_2_ (mEq/L), mean (SD)	20.1 (6.6)	20.4 (6.5)	19.3 (6.6)	0.034
AST (U/L), median (IQR)	39.0 (26.0–74.0)	37.0 (25.0–70.0)	45.0 (28.0–91.0)	0.005
ALT (U/L), median (IQR)	20.0 (12.0–38.0)	20.0 (11.0–37.0)	19.0 (12.0–38.5)	0.914
Total bilirubin (mg/dL), median (IQR)	0.8 (0.6–1.4)	0.8 (0.6–1.4)	0.9 (0.6–1.5)	0.024
ALP (IU/L), median (IQR)	95.0 (71.8–132.0)	93.0 (71.0–125.0)	100.0 (76.0–148.0)	0.002
PT INR, median (IQR)	1.2 (1.1–1.4)	1.2 (1.1–1.4)	1.3 (1.2–1.5)	< 0.001
Troponin I (µg/L), mean (SD)	62.3 (570.2)	56.5 (598.6)	77.8 (485.9)	0.649
NT-proBNP, mean (SD)	7597.3 (10,760.1)	7527.8 (10,898.5)	7751.6 (10,445.0)	0.817
Treatment				
Steroid, n (%)	564 (69.1%)	385 (64.9%)	179 (80.3%)	< 0.001
Vasopressor, n (%)	353 (43.3%)	195 (32.9%)	158 (70.9%)	< 0.001

APACHE, Acute Physiology and Chronic Health Evaluation; ALP, alkaline phosphatase; ALT, alanine aminotransferase; AST, aspartate aminotransferase; BUN, blood urea nitrogen; DBP, diastolic blood pressure; MBP, mean blood pressure; PR, pulse rate; RR, respiratory rate; SBP, systolic blood pressure; NT-proBNP, n-terminal prohormone brain natriuretic peptide; PT INR, international normalized ratio of prothrombin time; SAPS, Simplified Acute Physiology Score; SOFA, Sequential Organ Failure Assessment.

### Overall performance of the prediction models

**Figure 2:**
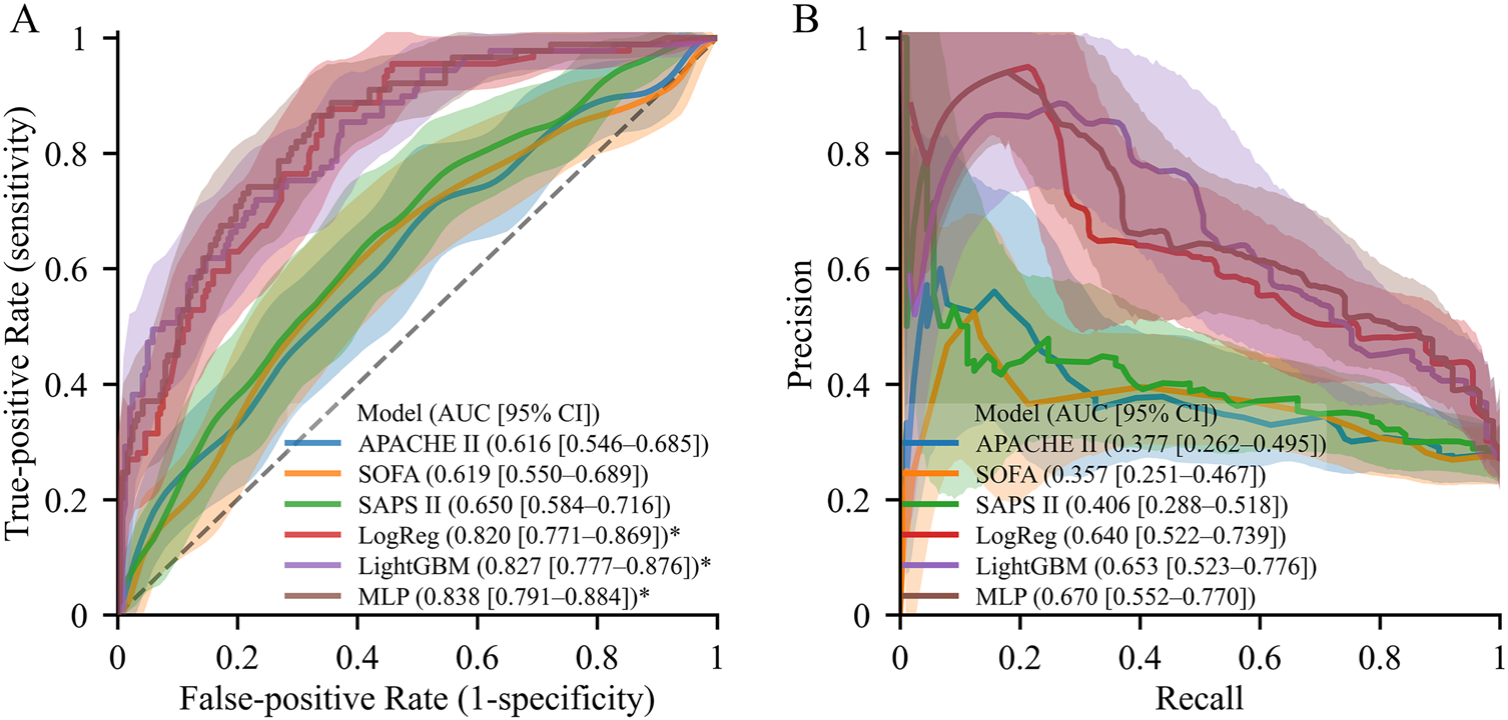
Overall performance of the models. (**A**) Receiver operating characteristics curves and (**B**) precision-recall curves. Solid lines and shades indicate the mean curves and 95% confidence interval areas, respectively. SAPS II is the baseline model, and its confidence intervals are represented with a polka dot pattern. An asterisk (*) indicates a significantly higher area under the receiver operating characteristics curve than the baseline model (*P* < 0.05, Benjamini–Hochberg corrected).

The prognostic performance and accuracy of the prediction models for in-ICU mortality are summarized in Fig. 2. Among the conventional severity-of-illness scoring systems, AU-ROC and AU-PRC were numerically (not statistically) higher in SAPS II than other conventional clinical scoring systems (SAPS II, AU-ROC (0.650 [95% CI, 0.584–0.761]) and AU-PRC (0.406 [0.288–0.518]); SOFA (AU-ROC, 0.619 [0.550–0.689] and AU-PRC, 0.357 [95% CI, 0.251–0.467]), and APACHE II (AU-ROC, 0.616 [95% CI, 0.546–0.685] and AU-PRC, 0.377 [95% CI, 0.262–0.495]) (Supplementary Table S5).

All the proposed ML models significantly outperformed all scoring systems with respect to AU-ROC (*P* < 0.001). The MLP model showed better performance than SAPS II, with an AU-ROC of 0.838 [0.791–0.884]), although there was no significant difference compared with the logistic regression (0.820 [0.771–0.869]) and LightGBM (0.827 [0.777–0.876]) models. Furthermore, all tested ML models showed numerically higher AU-PRC values for predicting in-ICU mortality than the SAPS II. The MLP model showed the highest AU-PRC value of 0.670 (95% CI, 0.552–0.770), which was numerically higher than that of the logistic regression (0.640 [0.522–0.739]) and LightGBM (0.653 [0.552–0.770]) models, while SAPS II dropped around 0.406. The cross-validation performance results are presented in Supplementary Table S6. The calibration and decision curves are shown in Supplementary Figs. S2 and S3, respectively. The detailed performance and NRI at the 10%, 20%, and 30% FPR levels are presented in Supplementary Table S7.

### Overall performance of ML models according to subgroups

**Figure 3:**
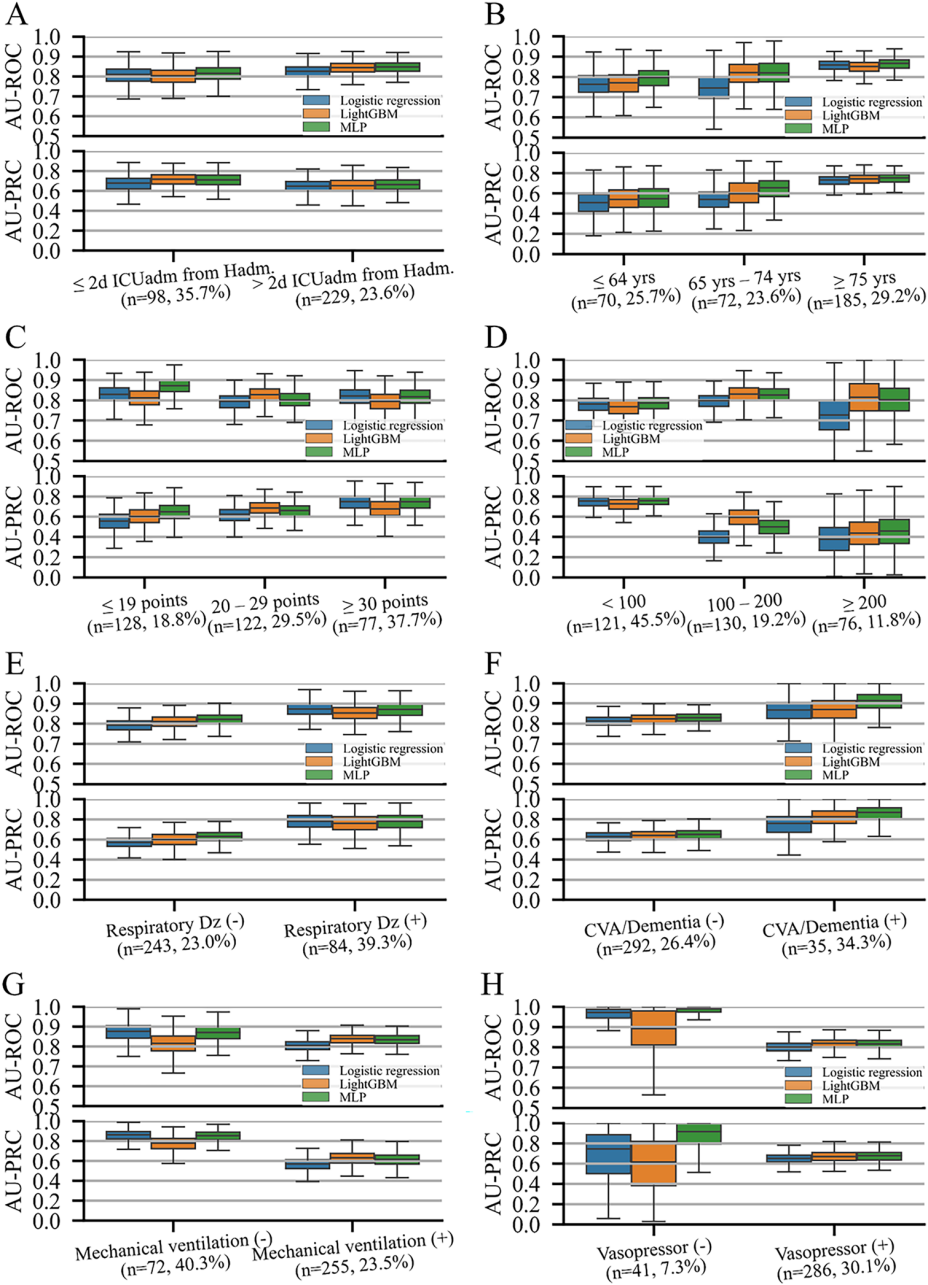
Overall performance by subgroup. The area under the receiver operating characteristic curves and precision-recall curves. The notation (n = a, b%) under each subgroup name indicates (**A**) the number of samples in the test set and (**B**) the prevalence rate of the outcome of the subgroup. Box plots are plotted with whiskers of 1.5 times the interquartile ranges. The subgrouping variables are (**A**) the period from hospital admission to ICU admission, (**B**) age, (**C**) Acute Physiology and Chronic Health Evaluation (APACH) II scores, (**D**) PaO_2_/FiO_2_ ratio, (**E**) history of chronic respiratory disease, (**F**) history of cerebrovascular accident or dementia, (**G**) mechanical ventilation, and (**H**) vasopressor use. AU-PRC, area under the precision-recall curve; AU-ROC, area under the receiver operating characteristics curve; CVA, cerebrovascular accident; Dz, disease; Hadm, hospital admission; ICU admission; yrs, years.

Figure 3 shows the overall performance of the ML models for in-ICU mortality in the different subgroups. The models performed consistently in most of the subgroups. Despite a difference in the in-ICU mortality rate, there was no significant difference in AU-ROC between the models in most of the subgroups except for the age 65–74 years subgroup (logistic regression vs MLP; *P* = 0.023), APACHE II score ≤ 19 subgroup (logistic regression vs MLP; *P* = 0.045, LightGBM vs MLP; *P* = 0.031), and MV treatment subgroup (logistic regression vs MLP; *P* = 0.044). There was no significant difference in the performance of the ML models in other subgroups.

In the analysis for NRI, the LightGBM and MLP models showed superior reclassification compared with the logistic regression model in predicting in-ICU mortality in all LOS in the ICU subgroups; all age subgroups; all subgroups with any APACHE II score, PaO_2_/FiO_2_ ratio < 200; all subgroups with or without history of respiratory disease; with or without history of CVA or dementia; treatment with MV, and use of inotropic agents (Supplementary Table S8). In most of the above subgroups, the LightGBM model showed higher NRI values than the MLP except for the subgroups aged 65–74 years, with an APACHE II score of ≤ 19, with history of CVA or dementia.

### Attributable variables with importance plots and SHAP values

**Figure 4:**
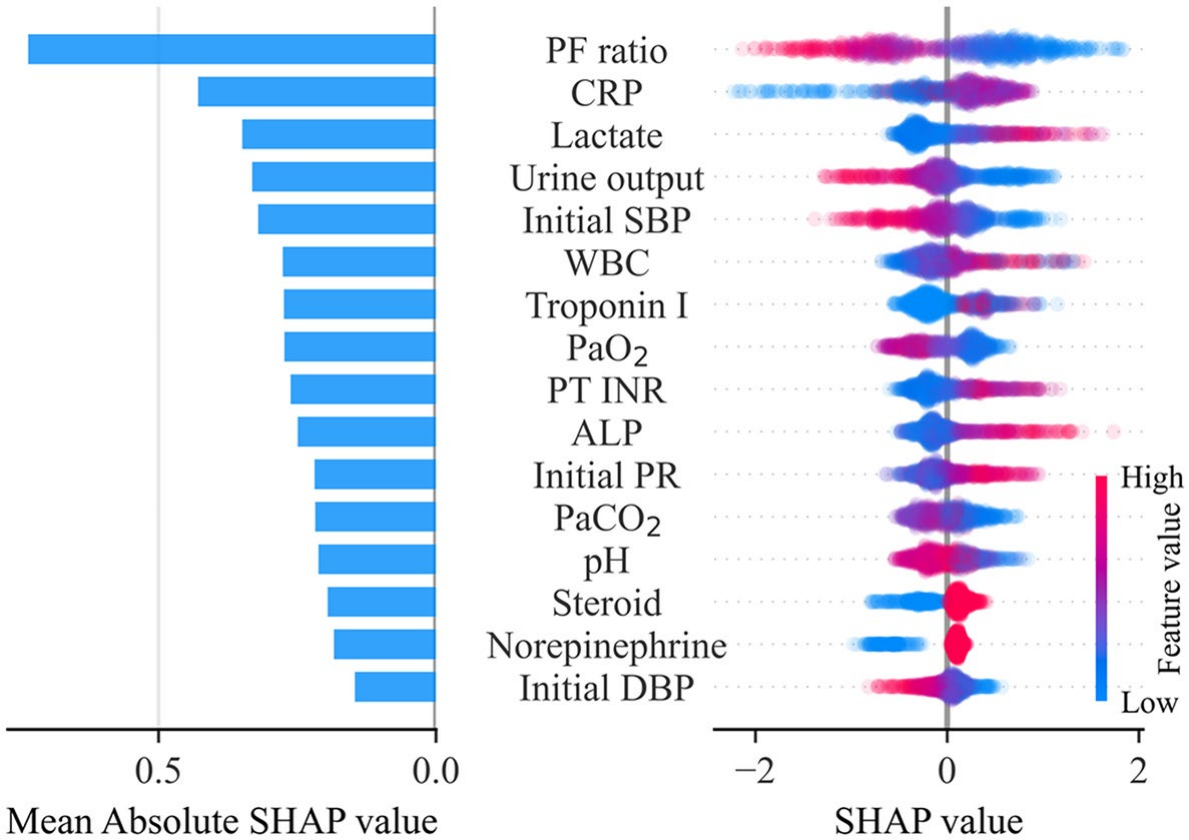
Importance of the selected variables. The individual influences of every value and the overall contributions of each variable to the model prediction are represented as a dot and a bar on the right and left, respectively. In the plot on the right, the red dots indicate high feature values for continuous/ordinal variables or affirmative responses for binary variables. Positive and negative SHAP values indicate that positive contributions result in an increased prediction score and that negative contributions result in a decreased prediction score. ALP, alkaline phosphatase; CRP, C-reactive protein; DBP, diastolic blood pressure; PF ratio, PaO_2_/FiO_2_ ratio; PR, pulse rate; PT-INR, international normalized ratio of prothrombin time; SBP, systolic blood pressure; WBC, white blood cell.

The selected predictors of in-ICU mortality are shown in Fig. 4. From a total of 55 variables, 16 were selected. The selected variables were PaO_2_/FiO_2_ ratio, CRP level, lactate level, urine output, initial systolic blood pressure (SBP), white blood cell (WBC) count. Among these variables, the partial SHAP dependence plots for the top six variables with the mean absolute SHAP values are illustrated in Supplementary Fig. S4; those for the other variables in Supplementary Fig. S5. The local interpretability of the LightGBM model is demonstrated in Supplementary Fig. S6, which shows how the model predicts each case of true positive, true negative, false positive, and false negative.

## Discussion

Our study evaluated the prognostic accuracy of ML models compared with that of conventional severity-of-illness scoring systems for predicting in-ICU mortality in patients with severe pneumonia. All ML models showed excellent performance in predicting in-ICU mortality and were superior to SAPS II. In addition, when the ML models were applied in the different subgroups, the LightGBM and MLP models showed superior reclassification compared with the logistic regression model in predicting in-ICU mortality in all LOS in the ICU subgroups; all age subgroups; all subgroups with any APACHE II score, PaO_2_/FiO_2_ ratio < 200; all subgroups with or without history of respiratory disease; with or without history of CVA or dementia; treatment with MV, and use of inotropic agents. Furthermore, the LightGBM model showed higher NRI values than the MLP in most of the above subgroups, except for the subgroups aged 65–74 years, with an APACHE II score of ≤ 19, with history of CVA or dementia. Therefore, ML models have the potential to improve in-ICU mortality prediction in patients with severe pneumonia admitted to the ICU. Moreover, this study shows the potential advantages of individual ML models for predicting in-ICU mortality in different subgroups of patients with severe pneumonia admitted to the ICU.

CURB-65 and PSI are the most commonly used clinical severity-of-illness scoring systems for patients with community-acquired pneumonia. However, CURB-65 and PSI have a limitation in that CURB-65 has low sensitivity, while PSI has low specificity for mortality^54^. The Clinical Pulmonary Infection Score is a well-validated prediction model for the development of ventilator-associated pneumonia. However, its predictive performance for mortality is inferior to that of the APACHE II score^55^. Given the limitations of clinical severity-of-illness scoring systems, the usefulness of ML models has recently been studied to predict mortality in patients with pneumonia. The results show that various ML models outperform CURB-65^38,39,56^ and PSI^57^ for predicting mortality in patients with severe community- or hospital-acquired pneumonia. However, few previous studies have used ML models to predict the prognosis of patients with severe pneumonia admitted to the ICU. One small study reported that an ML approach had better performance than APACHE II and PSI for predicting mortality in critically ill influenza patients^58^. In our study, SAPS II showed a numerically higher AU-ROC than APACHE II and SOFA for predicting in-ICU mortality on the first day of ICU admission. Importantly, the ML models outperformed SAPS II, which suggested that the ML model can provide more accurate information for optimal decision-making based on the estimated probability of mortality.

We selected logistic regression, LightGBM, and MLP as the ML predictive models for mortality in patients with severe pneumonia based on several considerations. Logistic regression is a well-established and widely used ML model for binary classification tasks. It offers a simple and interpretable approach to modeling the relationship between predictor variables and the outcome as well as performance of ML^59–61^. LightGBM is a gradient-boosted decision tree algorithm that has gained popularity for its high performance and efficiency^62^. Several studies have demonstrated the favorable predictive value of LightGBM in the field of medicine^63–65^. Another study found that LightGBM had the best predictive ability among other ML models including XGBoost, logistic regression, and naïve Bayes^66^. MLP-based models are effective in capturing nonlinear relationships, making them ideal candidates for complex and multifactorial disease classification including in stroke^67,68^ when compared to conventional statistical modeling. Moreover, both LightGBM and MLP have been used in many clinical studies^69–71^, demonstrating their extensive applicability and promising predictive performance. In the present study, the ML models outperformed conventional severity-of-illness systems. Scoring systems use a limited number of variables, which might restrict their predictive power in individual patients^72^. ML models are capable of utilizing high-dimensional data, and this could account for their superior performance to conventional scoring systems.

In this study, the LightGBM model showed the highest predictive performance with respect to NRI at 10% FPR. This result supports that decision-tree-based models could be more beneficial than logistic regression models for predicting in-ICU mortality in pneumonia patients at a high cut-off point of 90% specificity. ML models have a strength in capturing the nonlinear relations between the features and the predicted outcomes. We found notable non-linear relationships between in-ICU mortality and several selected variables, including PaO_2_, WBC count, pH, initial pulse rate, lymphocyte, HCO_3−_, and PaCO_2_. This could be the reason for the lower performance of the logistic regression model, a generalized linear model, in predicting the in-ICU mortality of patients with severe pneumonia. Although cross-validation results were not specified to the FPR level, the MLP model had the largest difference in the AU-ROC value between internal validation and cross-validation. This indicates that compared with the other models, the MLP model might be more relatively overfitted to the training set.

Contrary to the performance in different subgroups at the 10% FPR level, our results in the entire test set demonstrated no significant difference in AU-ROC between the ML models and the logistic regression model. In partial dependence plots of the variables that contributed the most to model predictions, linear relationships with in-ICU mortality were observed, especially in those of the PaO_2_/FiO_2_ ratio, urine output, and initial SBP. This might be the reason for the lack of statistical significance in these differences. The SHAP model was used to determine the important influencing factors of in-ICU mortality in the ML models, and the results were similar to previous studies: PaO_2_/FiO_2_^73,74^, CRP levels^75^, urine output^76^, initial SBP^73^, PaO_2_^73,77^, and leukopenia^78,79^ or leukocytosis^80,81^.

In addition, using the SHAP model, we found that higher ALP levels and prolonged PT-INR were associated with a higher risk of in-ICU mortality. ALP can be elevated as an acute-phase reaction in acute infections^82^. In community-acquired pneumonia, elevated ALP levels were not associated with mortality^83^. However, in critically ill patients with septic acute kidney injury (AKI), elevated ALP levels are associated with mortality^84^. In our study, septic shock was found in > 40% of the critically ill patients with pneumonia, and AKI was a common condition. It appears that ALP is related to the severity of impaired renal function through systemic inflammation caused by pneumonia rather than the severity of pneumonia itself. With respect to prolonged PT-INR, substantial coagulation abnormalities are commonly observed in patients with sepsis or pneumonia^85^. The excessive production of thrombogenic tissue factors in sepsis pneumonia compared with low levels of tissue factors under normal conditions^86^ leads to the development of systemic coagulopathy during the period of pneumonia^87^.

Our study had some limitations. First, as the models were developed using data retrospectively collected in a single center and were not externally validated, the results had limited generalizability. Although our study provides valuable insights into the performance of the ML models, further studies are needed to assess the generalizability and real-time applicability of these ML models in predicting in-ICU mortality in patients with severe pneumonia. Furthermore, these studies should include robust external validation using independent datasets and evaluation of the model performance in prospective clinical practice. Second, owing to the small sample size and the inclusion of patients admitted within a long study period of over 6 years, there is a possibility of heterogeneity with respect to patient characteristics, treatment measures, and potential biases. Thus, it might be challenging to clearly establish the usefulness of ML, which is a non-parametric algorithm. Furthermore, six subgrouping variables were adopted. A large number of stratification variables with a small sample size could lead to optimistic results in the internal validation of the models.

## Conclusion

Compared to conventional severity-of-illness scoring systems, the ML models of LightGBM, MLP, and logistic regression have better predictive performance for in-ICU mortality in patients with severe pneumonia. Moreover, this study shows the potential advantages of selecting individual ML models for predicting in-ICU mortality in different subgroups of patients with severe pneumonia.

## Supplementary Information

Supplementary Information.

## Data Availability

The datasets used and/or analyzed during the current study are available from the corresponding author upon reasonable request.
