# Supplementary material for: Machine learning-based prediction of in-ICU mortality in pneumonia patients
Source: Sci Rep. 2023 Jul 17;13:11527. doi: 10.1038/s41598-023-38765-8 (PMC10352276; doi:10.1038/s41598-023-38765-8)
Supplement: Supplementary file 1 — Supplementary Information. [file 41598_2023_38765_MOESM1_ESM.docx]

# **Supplementary materials**

**Machine Learning-Based Prediction of In-ICU Mortality for Pneumonia Patients Admitting to Intensive Care Unit**

**Supplementary Methods**

**Supplementary Tables**

Table S1. Included variables with their type and missing rate

Table S2. Hyperparameter tuning results

Table S3. Comparison of general characteristics between the training set and the test set

Table S4. Comparison of clinical features between the training set and the test set

Table S5. Test set performance of the scoring systems

Table S6. Cross-validation performance

Table S7. Detailed performance and net reclassification improvement at specific levels of false positive rate

Table S8. Detailed performance and net reclassification improvement at 10% false positive rate in different subgroups

**Supplementary Figures**

Figure S1. Machine learning workflow diagram

Figure S2. Calibration curves before and after calibration of the models

Figure S3. Decision curves for scoring systems and tested models

Figure S4. Partial SHAP dependence plots for top-six important variables

Figure S5. Partial SHAP dependence plots for the selected variables

Figure S6. Local interpretability of the developed gradient-boosted tree-based model

**Supplementary Methods**

**Modeling libraries and hyperparameter settings**

We tested one conventional statistical model and two popular machine learning models for the outcome prediction. The statistical model was logistic regression, and the model was developed using the class LogisticRegression from the scikit-learn library (version 0.22.1). Among the arguments, the solver and penalty were set as ‘lbfgs’ and ‘l2’, respectively. The left arguments were remained as default setting except C which is an inverse of regularization strength. The value of argument C was selected using Bayesian optimization by maximizing the area under the receiver operating characteristic curve (AU-ROC) on the validation set.

The machine learning models were LightGBM and multi-layer perceptron (MLP). LightGBM was developed using the class LGBMClassifier from the LightGBM library (version 3.3.2).^1^ Among the arguments, num_leaves (Maximum tree leaves), n_estimators (Number of boosted trees), min_split_gain (Minimum loss reduction required to make a further partition on a leaf node of the tree), min_child_weight (Minimum sum of instance weight needed in a leaf), min_child_samples (Minimum number of data needed in a leaf), and reg_lambda (L2 regularization term on weights) were tuned using Bayesian optimization by maximizing AU-ROC on the validation set. The model training was stropped when there is no improvement in the AU-ROC on the validation set over 10 consecutive training iterations. The left arguments were remained as default setting.

MLP was developed using the class Sequential and the class Layer in the Keras module from the tensorflow-gpu library (version 2.4.0). We consisted of the base architecture with an input layer, a hidden layer with 64 nodes, a batch normalization layer, a drop-out layer, and an output layer, sequentially. The optimizer was Nadam (Adam optimizer with Nesterov technique) and learning rate was set as 0.005. The activation function was ELU (the Exponential Linear Unit). We determined additional depth following the existing hidden layer, argument momentum of BatchNormalization function, rate of the drop-out layer, and l of l2 function in the hidden layers using Bayesian optimization by maximizing AU-ROC on the validation set. The additional depth was a bunch of sets, and each set was consisted of a dense layer with 32 nodes and a batch normalization layer. The model training was stropped when there is no improvement in the AU-ROC on the validation set over 20 consecutive training iterations. The left arguments were remained as default setting.

The search spaces and selected values for the hyperparameters were represented in **Table S2**.

^1^Guolin Ke, Qi Meng, Thomas Finley, Taifeng Wang, Wei Chen, Weidong Ma, Qiwei Ye, Tie-Yan Liu. "LightGBM: A Highly Efficient Gradient Boosting Decision Tree". Advances in Neural Information Processing Systems 30 (NIPS 2017), pp. 3149-3157.

**Supplementary Tables**

| **Table S1.** Included variables with their type and missing rate. | | | | |  |
| --- | --- | --- | --- | --- | --- |
| **Variable** | | **Variable type** | | **Missing rate, %** |  |
| **Demographics** | |  | |  |  |
| Age | | Continuous | | 0 |  |
| Sex | | Categorical | | 0 |  |
| Smoking* | | Categorical | | 0 |  |
| Pack years | | Continuous | | 3.7 |  |
| **Underlying comorbidities** | |  | |  |  |
| Hypertension | | Categorical | | 0 |  |
| Diabetes | | Categorical | | 0 |  |
| COPD | | Categorical | | 0 |  |
| Asthma | | Categorical | | 0 |  |
| Interstitial lung disease | | Categorical | | 0 |  |
| Nontuberculous mycobacteria | | Categorical | | 0 |  |
| Tuberculosis | | Categorical | | 0 |  |
| Lung cancer | | Categorical | | 0 |  |
| Chronic kidney disease | | Categorical | | 0 |  |
| Chronic liver disease | | Categorical | | 0 |  |
| Cardiovascular disease | | Categorical | | 0 |  |
| Congestive heart failure | | Categorical | | 0 |  |
| Cerebrovascular accident | | Categorical | | 0 |  |
| Dementia | | Categorical | | 0 |  |
| Metastatic cancer | | Categorical | | 0 |  |
| **Initial vital signs** | |  | |  |  |
| Glasgow Coma Scale | | Continuous | | 0.1 |  |
| Initial systolic blood pressure | | Continuous | | 0 |  |
| Initial diastolic blood pressure | | Continuous | | 0 |  |
| Initial pulse rate | | Continuous | | 0 |  |
| Initial respiratory rate | | Continuous | | 0 |  |
| Body temperature | | Continuous | | 0.6 |  |
| Urine output (per hour) | | Continuous | | 0.5 |  |
| **Laboratory findings** | |  | |  |  |
| pH | | Continuous | | 0 |  |
| PaCO_2_ | | Continuous | | 0 |  |
| PaO_2_ | | Continuous | | 0 |  |
| PaO_2_/FiO_2_ ratio | | Continuous | | 0.1 |  |
| White blood cell | | Continuous | | 0 |  |
| Hemoglobin | | Continuous | | 0 |  |
| Platelet | | Continuous | | 0 |  |
| Lactate | | Continuous | | 15.4 |  |
| C-reactive protein | | Continuous | | 0.1 |  |
| Blood urea nitrogen | | Continuous | | 0 |  |
| Creatinine | | Continuous | | 0 |  |
| Na | | Continuous | | 0 |  |
| K | | Continuous | | 0 |  |
| tCO2 | | Continuous | | 0.9 |  |
| Aspartate aminotransferase | | Continuous | | 0 |  |
| Alanine aminotransferase | | Continuous | | 0 |  |
| **Table S1.** (*Continued.*) | | | | | |
| **Variable** | **Variable type** | | **Missing rate, %** | | |
| Total bilirubin | Continuous | | 0 | | |
| Alkaline phosphatase | Continuous | | 0 | | |
| PT INR | Continuous | | 0.2 | | |
| Troponin I | Continuous | | 7.8 | | |
| Hematocrit | Continuous | | 0 | | |
| HCO_3_ | Continuous | | 0 | | |
| Neutrophil | Continuous | | 0 | | |
| Lymphocyte | Continuous | | 0 | | |
| **Treatment** |  | |  | | |
| Steroid | Categorical | | 0 | | |
| Antibiotics | Categorical | | 0 | | |
| Norepinephrine | Categorical | | 0 | | |
| Dopamine | Categorical | | 0 | | |
| *implies categorical variables having more than 3 categories. Smoking was grouped into non-smoker, ex-smoker, and current smoker. Abbreviations: PT INR, international normalized ratio of prothrombin time. | | | | | |

| **Table S2.** Hyperparameter tuning results | | | | | |
| --- | --- | --- | --- | --- | --- |
|  | **Model** | **Hyperparameter** | **Type** | **Search space (min, max)** | **Selected value** |
|  | Logistic regression | C | float | (0.1, 10) | 0.9521 |
|  | LightGBM | num_leaves | integer | (40, 130) | 118 |
|  |  | n_estimators | integer | (70, 300) | 259 |
|  |  | min_split_gain | float | (0.001, 0.1) | 0.0261 |
|  |  | min_child_weight | float | (0, 0.02) | 0.0021 |
|  |  | min_child_samples | integer | (2, 50) | 43 |
|  |  | reg_lambda | float | (0, 0.3) | 0.2565 |
|  | Multi-layer perceptron | additional depth† | integer | (0, 3) | 2 |
|  |  | BN momentum | float | (0.8, 0.99) | 0.8335 |
|  |  | drop-out rate | float | (0.5 0.7) | 0.5431 |
|  |  | l2 regularization strength | float | (0, 0.4) | 0.3011 |
| †This means the number of additional sets and each set consisted of a dense layer with 32 nodes and a batch normalization layer. The additional sets positioned between the existing hidden layer and the drop-out layer. Abbreviation: BN, batch normalization. | | | | | |

| **Table S3. Comparison of general characteristics between the training set and the test set** | | | | |
| --- | --- | --- | --- | --- |
| **Variable** | **All** | **Training set** | **Test set** | ***P* value** |
|  | **(n = 816)** | **(n = 489, 59.9 %)** | **(n = 327, 40.1 %)** |  |
| **Demographics** | | | | |
| Age, yrs, median (IQR) | 77 (67 – 83) | 77 (66 – 83) | 77 (67 – 83) | 0.774 |
| Male, n (%) | 588 (72.1 %) | 357 (73.0 %) | 231 (70.6 %) | 0.511 |
| BMI, kg/m^2^, mean (SD) | 20.7 (4.5) | 20.6 (4.3) | 20.7 (4.7) | 0.727 |
| Smoking |  |  |  | 0.652 |
| Ex-smoker, n (%) | 233 (28.6 %) | 140 (28.6 %) | 93 (28.4 %) |  |
| Current smoker, n (%) | 43 (5.3 %) | 28 (5.7 %) | 15 (4.6 %) |  |
| Pack years in ever-smokers, mean (SD) | 9.9 (19.0) | 9.8 (18.3) | 10.0 (19.8) | 0.899 |
| **Underlying comorbidities** | | | | |
| Hypertension, n (%) | 450 (55.1 %) | 272 (55.6 %) | 178 (54.4 %) | 0.793 |
| Diabetes, n (%) | 319 (39.1 %) | 189 (38.7 %) | 130 (39.8 %) | 0.807 |
| COPD, n (%) | 62 (7.6 %) | 36 (7.4 %) | 26 (8.0 %) | 0.860 |
| Asthma, n (%) | 24 (2.9 %) | 15 (3.1 %) | 9 (2.8 %) | 0.960 |
| Interstitial lung disease, n (%) | 31 (3.8 %) | 23 (4.7 %) | 8 (2.4 %) | 0.143 |
| Nontuberculous mycobacteria, n (%) | 8 (1.0 %) | 6 (1.2 %) | 2 (0.6 %) | 0.609 |
| Tuberculosis, n (%) | 119 (14.6 %) | 70 (14.3 %) | 49 (15.0 %) | 0.869 |
| Lung cancer, n (%) | 11 (1.3 %) | 7 (1.4 %) | 4 (1.2 %) | 1.000 |
| Chronic kidney disease, n (%) | 121 (14.8 %) | 59 (12.1 %) | 62 (19.0 %) | 0.009 |
| Chronic liver disease, n (%) | 48 (5.9 %) | 28 (5.7 %) | 20 (6.1 %) | 0.936 |
| Cardiovascular disease, n (%) | 14 (1.7 %) | 11 (2.2 %) | 3 (0.9 %) | 0.246 |
| Congestive heart failure, n (%) | 32 (3.9 %) | 17 (3.5 %) | 15 (4.6 %) | 0.537 |
| Cerebrovascular accident, n (%) | 11 (1.3 %) | 5 (1.0 %) | 6 (1.8 %) | 0.499 |
| Dementia, n (%) | 63 (7.7 %) | 34 (7.0 %) | 29 (8.9 %) | 0.384 |
| HIV, n (%) | 5 (0.6 %) | 4 (0.8 %) | 1 (0.3 %) | 0.645 |
| Metastatic cancer, n (%) | 11 (1.3 %) | 7 (1.4 %) | 4 (1.2 %) | 1.000 |
| Hematologic malignancy, n (%) | 2 (0.2 %) | 2 (0.4 %) | 0 (0.0 %) | 0.663 |
| Abbreviations: BMI, body mass index; COPD, chronic obstructive pulmonary disease; HIV, human immunodeficiency virus. | | | | |

| **Table S4. Comparison of clinical features between the training set and the test set** | | | | |
| --- | --- | --- | --- | --- |
| **Variable** | **All** | **Training set** | **Test set** | ***P* value** |
|  | **(n = 816)** | **(n = 489, 59.9 %)** | **(n = 327, 40.1 %)** |  |
| **Prognostic scores** | | | | |
| APACHE II | 21 (16 – 28) | 21 (16 – 27) | 22 (16 – 28) | 0.523 |
| SOFA | 10 (7 – 13) | 10 (7 – 13) | 10 (7 – 13) | 0.378 |
| SAPS II | 48 (37 – 62) | 48 (36 – 61) | 48 (37 – 63) | 0.321 |
| **Initial vital signs** | | | | |
| GCS | 11 (6 – 14) | 11 (6 – 14) | 10 (6 – 14) | 0.523 |
| Initial SBP, mmHg | 122.0 (102.0 – 146.0) | 123.0 (104.0 – 145.0) | 122.0 (100.0 – 148.0) | 0.808 |
| Initial DBP, mmHg | 64.0 (54.0 – 75.2) | 64.0 (54.0 – 75.0) | 64.0 (52.0 – 76.0) | 0.833 |
| Initial MBP | 84.3 (70.7 – 98.0) | 84.3 (71.3 – 98.0) | 84.3 (69.3 – 96.8) | 0.911 |
| Initial PR, beats per minute | 104.0 (86.0 – 120.0) | 103.0 (86.0 – 120.0) | 104.0 (89.0 – 121.0) | 0.666 |
| Initial RR, breaths per minute | 22.0 (19.0 – 27.0) | 23.0 (20.0 – 26.0) | 22.0 (19.0 – 28.0) | 0.892 |
| Body temperature, °C, mean (SD) | 36.9 (1.1) | 36.9 (1.1) | 36.8 (1.1) | 0.352 |
| Urine output, ml/hour, mean (SD) | 59.9 (30.0 – 91.7) | 57.8 (29.4 – 88.3) | 60.7 (31.7 – 95.7) | 0.570 |
| **Laboratory findings** | | | | |
| pH, median (IQR) | 7.4 (7.3 – 7.5) | 7.4 (7.3 – 7.5) | 7.4 (7.3 – 7.4) | 0.496 |
| pCO_2_, mmHg, median (IQR) | 35.3 (29.2 – 43.7) | 34.9 (29.0 – 42.8) | 35.6 (29.3 – 44.6) | 0.123 |
| pO_2_, mmHg, median (IQR) | 68.3 (54.0 – 85.7) | 66.5 (53.4 – 84.9) | 70.6 (57.0 – 86.2) | 0.177 |
| HCO_3_, mEq/L, median (IQR) | 21.0 (17.1 – 24.7) | 21.0 (16.9 – 24.8) | 21.1 (17.4 – 24.5) | 0.442 |
| Initial SpO_2_, median (IQR) | 95.0 (90.0 – 98.0) | 95.0 (90.0 – 98.0) | 95.0 (91.0 – 98.0) | 0.666 |
| pO_2_/FiO_2_ ratio, median (IQR) | 121.6 (78.3 – 194.0) | 116.2 (76.8 – 194.6) | 132.5 (80.3 – 193.2) | 0.174 |
| White blood cell, 10^3^/μL, median (IQR) | 11.2 (7.6 – 16.3) | 11.5 (7.7 – 16.9) | 11.0 (7.4 – 15.5) | 0.247 |
| Neutrophil, 10^3^/μL, median (IQR) | 84.0 (75.9 – 89.2) | 84.1 (75.8 – 89.4) | 83.8 (76.0 – 89.2) | 0.673 |
| Lymphocyte, 10^3^/μL, median (IQR) | 10.1 (6.6 – 16.0) | 10.0 (6.3 – 16.1) | 10.6 (7.0 – 15.8) | 0.244 |
| Hemoglobin, median (IQR) | 10.9 (9.2 – 12.5) | 10.9 (9.4 – 12.5) | 10.9 (9.0 – 12.5) | 0.561 |
| Platelet, 10^3^/μL, median (IQR) | 205.0 (134.0 – 294.2) | 205.0 (136.0 – 295.0) | 201.0 (129.0 – 293.5) | 0.593 |
| Lactate, mmol/L | 4.3 (5.2) | 4.3 (5.4) | 4.3 (4.7) | 0.967 |
| C-reactive protein, mg/L, median (IQR) | 13.4 (5.8 – 22.0) | 13.3 (5.7 – 22.3) | 13.6 (5.8 – 21.9) | 0.990 |
| Procalcitonin, ng/mL, mean (SD) | 2.4 (7.7) | 2.0 (6.4) | 3.2 (9.6) | 0.194 |
| BUN, mg/dL, median (IQR) | 27.0 (15.0 – 44.0) | 27.0 (15.0 – 43.0) | 27.0 (15.0 – 45.5) | 0.632 |
| Creatinine, mg/dL, median (IQR) | 1.2 (0.8 – 2.1) | 1.2 (0.8 – 1.9) | 1.2 (0.8 – 2.3) | 0.978 |
| Na, mEq/L, median (IQR) | 136.0 (132.2 – 139.8) | 136.0 (132.8 – 139.9) | 136.0 (131.5 – 139.2) | 0.201 |
| K, mEq/L, median (IQR) | 4.1 (3.6 – 4.7) | 4.1 (3.5 – 4.7) | 4.1 (3.6 – 4.6) | 0.657 |
| TCO_2_, mEq/L, mean (SD) | 20.1 (6.6) | 19.9 (6.2) | 20.4 (7.0) | 0.268 |
| AST, U/L, median (IQR) | 39.0 (26.0 – 74.0) | 40.0 (26.0 – 77.0) | 38.0 (26.0 – 72.0) | 0.931 |
| ALT, U/L, median (IQR) | 20.0 (12.0 – 38.0) | 20.0 (11.0 – 36.0) | 19.0 (13.0 – 39.5) | 0.545 |
| Total bilirubin, mg/dL, median (IQR) | 0.8 (0.6 – 1.4) | 0.8 (0.6 – 1.4) | 0.9 (0.6 – 1.4) | 0.282 |
| ALP, IU/L, median (IQR) | 95.0 (71.8 – 132.0) | 94.0 (72.0 – 129.0) | 96.0 (71.5 – 135.5) | 0.430 |
| PT INR, median (IQR) | 1.2 (1.1 – 1.4) | 1.2 (1.1 – 1.4) | 1.2 (1.1 – 1.4) | 0.736 |
| Troponin I, µg/L, mean (SD) | 62.3 (570.2) | 74.8 (708.6) | 43.7 (249.3) | 0.463 |
| NT-proBNP, mean (SD) | 7597.3 (10760.1) | 7088.9 (10098.3) | 8385.8 (11668.8) | 0.157 |
| **Treatment** | | | | |
| Steroid, n (%) | 564 (69.1 %) | 334 (68.3 %) | 230 (70.3 %) | 0.590 |
| Vasopressor, n (%) | 353 (43.3 %) | 217 (44.4 %) | 136 (41.6 %) | 0.475 |
| Abbreviations: APACHE, the Acute Physiology and Chronic Health Evaluation; ALP, Alkaline phosphatase; ALT, Alanine aminotransferase; AST, aspartate aminotransferase; BUN, Blood urea nitrogen; DBP, diastolic blood pressure; MBP, mean blood pressure; PR, pulse rate; RR, respiratory rate; SBP, systolic blood pressure; NT-proBNP, n-terminal prohormone brain natriuretic peptide; PT INR, international normalized ratio of prothrombin time; SAPS, the Simplified Acute Physiology Score; SOFA, the Sequential Organ Failure Assessment. | | | | |

| **Table S5. Test set performance of the scoring systems** | | | | | | | | |
| --- | --- | --- | --- | --- | --- | --- | --- | --- |
| **Model** | **AU-ROC** | **AU-PRC** | **Brier score** | **ACC, %** | **Precision** | **Recall** | **F1 score** |  |
| APACHE II | 0.616 (0.546 – 0.685) | 0.377 (0.262 – 0.495) | 0.193 | 73.1 | 0.571 | 0.045 | 0.083 |  |
| SOFA | 0.619 (0.550 – 0.689) | 0.357 (0.251 – 0.467) | 0.198 | 71.9 | 0 | 0 | 0 |  |
| SAPS II | 0.650 (0.584 – 0.716) | 0.406 (0.288 – 0.518) | 0.188 | 71.9 | 0.44 | 0.124 | 0.193 |  |
| AU-ROC and AU-PRC values are expressed as mean (95% CI). The values of other metrics are calculated with a cut-off threshold of 0.5. Abbreviations: AU-ROC, area under the receiver operating characteristic curve; AU-PRC, area under the precision-recall curve; ACC, accuracy. | | | | | | | | |

| **Table S6.** Cross-validation performance | | | | | | | |
| --- | --- | --- | --- | --- | --- | --- | --- |
| **Model** | **AU-ROC** | **AU-PRC** | **Brier score** | **ACC, %** | **Precision** | **Recall** | **F1 score** |
| LogReg | 0.832 (0.050) | 0.725 (0.066) | 0.130 (0.021) | 81.2 (3.4) | 0.74 (0.12) | 0.55 (0.15) | 0.60 (0.09) |
| LightGBM | 0.840 (0.047) | 0.732 (0.078) | 0.126 (0.022) | 82.0 (4.2) | 0.79 (0.16) | 0.52 (0.17) | 0.60 (0.12) |
| MLP | 0.856 (0.040) | 0.748 (0.072) | 0.120 (0.022) | 82.2 (4.1) | 0.86 (0.11) | 0.44 (0.17) | 0.56 (0.15) |
| The values are aggregated in all folds and expressed as mean (SD). Abbreviations: AU-ROC, area under the receiver operating characteristic curve; AU-PRC, area under the precision-recall curve; ACC, accuracy; LightGBM, light gradient-boosting machine; LogReg, logistic regression; MLP, multi-layer perceptron. | | | | | | | |

| **Table S7.** Detailed performance and net reclassification improvement at specific levels of false positive rate | | | | | |
| --- | --- | --- | --- | --- | --- |
|  | **Sensitivity** | **PPV** | **NPV** | **Diagnostic OR** | **NRI** |
| **At a false positive rate of 10%** | | | |  |  |
| SAPS II | 0.184 (0.077–0.292) | 0.434 (0.265–0.602) | 0.750 (0.699–0.801) | 2.475 (0.555–4.394) | Reference |
| LogReg | 0.415 (0.270–0.561) | 0.628 (0.516–0.739) | 0.807 (0.757–0.857) | 7.529 (2.786–12.272) | 0.231 (0.037–0.425) |
| LGBM | 0.515 (0.390–0.641) | 0.680 (0.602–0.757) | 0.835 (0.788–0.881) | 11.317 (5.111–17.523) | 0.310 (0.152–0.468) |
| MLP | 0.460 (0.305–0.616) | 0.647 (0.556–0.738) | 0.819 (0.764–0.874) | 8.893 (3.174–14.613) | 0.255 (0.083–0.426) |
|  |  |  |  |  |  |
| **At a false positive rate of 20%** | | | |  |  |
| SAPS II | 0.358 (0.255–0.461) | 0.420 (0.327–0.514) | 0.778 (0.726–0.829) | 2.613 (1.441–3.786) | Reference |
| LogReg | 0.618 (0.507–0.730) | 0.552 (0.466–0.638) | 0.854 (0.806–0.902) | 7.602 (3.524–11.680) | 0.244 (0.102–0.386) |
| LGBM | 0.659 (0.535–0.783) | 0.558 (0.481–0.635) | 0.866 (0.818–0.914) | 8.684 (3.569–13.798) | 0.253 (0.096–0.410) |
| MLP | 0.679 (0.561–0.797) | 0.565 (0.487–0.644) | 0.873 (0.828–0.918) | 9.511 (3.967–15.054) | 0.271 (0.117–0.426) |
|  |  |  |  |  |  |
| **At a false positive rate of 30%** | | | |  |  |
| SAPS II | 0.485 (0.352–0.618) | 0.393 (0.307–0.480) | 0.790 (0.732–0.849) | 2.546 (1.206–3.886) | Reference |
| LogReg | 0.755 (0.613–0.897) | 0.494 (0.420–0.569) | 0.887 (0.827–0.947) | 8.632 (1.466–15.799) | 0.280 (0.079–0.480) |
| LGBM | 0.743 (0.644–0.841) | 0.489 (0.423–0.556) | 0.881 (0.834–0.929) | 7.552 (3.313–11.792) | 0.281 (0.095–0.467) |
| MLP | 0.803 (0.694–0.912) | 0.508 (0.438–0.577) | 0.907 (0.856–0.958) | 11.173 (2.494–19.852) | 0.350 (0.153–0.547) |
| The values are expressed as mean (95% CI).  Abbreviations: LGBM, light gradient-boosting machine; LogReg, logistic regression; MLP, multi-layer perceptron; NPV, negative predictive value; NRI, net reclassification improvement; OR, odds ratio; PPV, positive predictive value; SAPS, the Simplified Acute Physiology Score. | | | | | |

| **Table S8.** Detailed performance and net reclassification improvement at 10% false positive rate in different subgroups | | | | | | |
| --- | --- | --- | --- | --- | --- | --- |
|  |  | **Sensitivity** | **PPV** | **NPV** | **Diagnostic OR** | **NRI** |
| **A period from hospital admission to ICU admission** | | | | |  |  |
| ≤ 2days | LogReg | 0.289 (0.068–0.510) | 0.689 (0.470–0.909) | 0.698 (0.591–0.804) | 1.614 (1.173–2.055) | Reference |
|  | LGBM | 0.397 (0.216–0.578) | 0.753 (0.601–0.905) | 0.730 (0.636–0.825) | 1.799 (1.437–2.161) | 0.102 (-0.103–0.307) |
|  | MLP | 0.334 (0.090–0.578) | 0.718 (0.526–0.910) | 0.711 (0.599–0.824) | 1.689 (1.216–2.162) | 0.041 (-0.117–0.199) |
| > 2 days | LogReg | 0.492 (0.302–0.682) | 0.622 (0.496–0.747) | 0.854 (0.793–0.916) | 2.102 (1.775–2.429) | Reference |
|  | LGBM | 0.587 (0.453–0.720) | 0.671 (0.581–0.761) | 0.879 (0.831–0.926) | 2.266 (2.030–2.502) | 0.097 (-0.065–0.260) |
|  | MLP | 0.539 (0.378–0.701) | 0.648 (0.540–0.756) | 0.866 (0.811–0.922) | 2.181 (1.904–2.458) | 0.048 (-0.078–0.174) |
| **Age** |  |  |  |  |  |  |
| ≤ 64 yrs | LogReg | 0.349 (0.089–0.608) | 0.703 (0.390–1.000) | 0.804 (0.698–0.910) | 1.995 (1.500–2.489) | Reference |
|  | LGBM | 0.420 (0.146–0.695) | 0.706 (0.405–1.000) | 0.821 (0.718–0.925) | 2.087 (1.572–2.602) | 0.047 (-0.113–0.208) |
|  | MLP | 0.378 (0.116–0.640) | 0.702 (0.383–1.000) | 0.811 (0.709–0.914) | 2.033 (1.545–2.521) | 0.020 (-0.115–0.156) |
| 65 yrs – 74 yrs | LogReg | 0.306 (0.028–0.584) | 0.599 (0.214–0.983) | 0.806 (0.709–0.904) | 1.915 (1.435–2.395) | Reference |
|  | LGBM | 0.498 (0.199–0.797) | 0.705 (0.479–0.932) | 0.853 (0.769–0.937) | 2.214 (1.721–2.707) | 0.245 (-0.104–0.594) |
|  | MLP | 0.518 (0.113–0.922) | 0.698 (0.463–0.933) | 0.860 (0.752–0.967) | 2.257 (1.595–2.919) | 0.269 (-0.128–0.667) |
| ≥ 75 yrs | LogReg | 0.497 (0.279–0.714) | 0.707 (0.587–0.828) | 0.815 (0.721–0.909) | 2.058 (1.619–2.498) | Reference |
|  | LGBM | 0.540 (0.379–0.701) | 0.722 (0.625–0.820) | 0.827 (0.751–0.904) | 2.132 (1.773–2.490) | 0.040 (-0.138–0.219) |
|  | MLP | 0.504 (0.335–0.674) | 0.693 (0.590–0.796) | 0.816 (0.738–0.894) | 2.040 (1.702–2.378) | 0.001 (-0.163–0.164) |
| **APACHE II scores** | |  |  |  |  |  |
| ≤ 19 points | LogReg | 0.422 (0.204–0.640) | 0.571 (0.354–0.787) | 0.871 (0.808–0.935) | 2.137 (1.803–2.471) | Reference |
|  | LGBM | 0.527 (0.340–0.715) | 0.624 (0.454–0.794) | 0.892 (0.836–0.948) | 2.279 (1.969–2.589) | 0.144 (-0.147–0.434) |
|  | MLP | 0.576 (0.323–0.829) | 0.632 (0.437–0.828) | 0.903 (0.845–0.962) | 2.349 (1.954–2.744) | 0.214 (-0.105–0.533) |
| 20 – 29 points | LogReg | 0.304 (0.110–0.498) | 0.606 (0.394–0.817) | 0.764 (0.675–0.854) | 1.757 (1.400–2.113) | Reference |
|  | LGBM | 0.415 (0.223–0.607) | 0.668 (0.506–0.831) | 0.794 (0.710–0.877) | 1.923 (1.571–2.275) | 0.160 (-0.102–0.423) |
|  | MLP | 0.334 (0.152–0.515) | 0.674 (0.475–0.874) | 0.774 (0.690–0.859) | 1.844 (1.493–2.194) | 0.056 (-0.183–0.295) |
| ≥ 30 points | LogReg | 0.492 (0.292–0.693) | 0.841 (0.703–0.979) | 0.756 (0.642–0.869) | 2.036 (1.532–2.539) | Reference |
|  | LGBM | 0.539 (0.272–0.805) | 0.852 (0.721–0.984) | 0.775 (0.661–0.890) | 2.155 (1.573–2.738) | 0.061 (-0.209–0.330) |
|  | MLP | 0.516 (0.277–0.754) | 0.809 (0.659–0.958) | 0.762 (0.635–0.889) | 2.038 (1.461–2.615) | 0.013 (-0.179–0.205) |

| **Table S8.** *(Continued.)* | | | | | | |
| --- | --- | --- | --- | --- | --- | --- |
|  |  | **Sensitivity** | **PPV** | **NPV** | **Diagnostic OR** | **NRI** |
| **PaO_2_/FiO_2_ ratio** | | | | | | |
| < 100 | LogReg | 0.408 (0.266–0.549) | 0.832 (0.708–0.956) | 0.655 (0.550–0.760) | 1.686 (1.326–2.045) | Reference |
|  | LGBM | 0.499 (0.317–0.680) | 0.834 (0.737–0.931) | 0.690 (0.568–0.811) | 1.856 (1.392–2.320) | 0.089 (-0.112–0.291) |
|  | MLP | 0.480 (0.307–0.653) | 0.832 (0.741–0.923) | 0.682 (0.562–0.801) | 1.815 (1.364–2.266) | 0.069 (-0.086–0.225) |
| 100 – 200 | LogReg | 0.246 (-0.093–0.585) | 0.372 (0.003–0.740) | 0.836 (0.757–0.916) | 1.874 (1.454–2.293) | Reference |
|  | LGBM | 0.415 (0.204–0.626) | 0.580 (0.389–0.772) | 0.869 (0.807–0.930) | 2.131 (1.803–2.458) | 0.186 (-0.152–0.525) |
|  | MLP | 0.413 (-0.031–0.856) | 0.498 (0.166–0.831) | 0.869 (0.776–0.962) | 2.089 (1.495–2.683) | 0.175 (-0.223–0.574) |
| ≥ 200 | LogReg | 0.407 (0.054–0.761) | 0.457 (0.108–0.806) | 0.920 (0.858–0.981) | 2.295 (1.880–2.709) | Reference |
|  | LGBM | 0.338 (-0.081–0.757) | 0.370 (0.031–0.710) | 0.910 (0.834–0.986) | 2.199 (1.709–2.689) | -0.117 (-0.879–0.644) |
|  | MLP | 0.409 (0.133–0.685) | 0.478 (0.146–0.809) | 0.920 (0.864–0.975) | 2.303 (1.929–2.677) | 0.006 (-0.441–0.454) |
| **History of chronic respiratory disease** | | |  |  |  |  |
| (-) | LogReg | 0.363 (0.207–0.520) | 0.549 (0.405–0.692) | 0.826 (0.776–0.876) | 1.912 (1.670–2.154) | Reference |
|  | LGBM | 0.449 (0.318–0.580) | 0.614 (0.509–0.719) | 0.846 (0.800–0.892) | 2.053 (1.823–2.283) | 0.072 (-0.041–0.184) |
|  | MLP | 0.439 (0.284–0.594) | 0.592 (0.479–0.705) | 0.843 (0.797–0.889) | 2.019 (1.792–2.246) | 0.059 (-0.045–0.163) |
| (+) | LogReg | 0.521 (0.244–0.797) | 0.827 (0.695–0.960) | 0.744 (0.610–0.878) | 2.021 (1.357–2.685) | Reference |
|  | LGBM | 0.597 (0.368–0.826) | 0.846 (0.732–0.960) | 0.774 (0.648–0.900) | 2.195 (1.565–2.826) | 0.056 (-0.116–0.227) |
|  | MLP | 0.532 (0.223–0.842) | 0.826 (0.669–0.983) | 0.749 (0.603–0.896) | 2.046 (1.301–2.791) | 0.005 (-0.173–0.183) |
| **History of cerebrovascular accident or dementia** | | | | |  |  |
| (-) | LogReg | 0.443 (0.271–0.615) | 0.632 (0.505–0.760) | 0.821 (0.768–0.874) | 1.980 (1.710–2.250) | Reference |
|  | LGBM | 0.505 (0.377–0.633) | 0.669 (0.574–0.763) | 0.837 (0.787–0.887) | 2.086 (1.860–2.311) | 0.067 (-0.085–0.218) |
|  | MLP | 0.447 (0.280–0.614) | 0.627 (0.511–0.743) | 0.822 (0.766–0.877) | 1.973 (1.708–2.238) | 0.000 (-0.135–0.136) |
| (+) | LogReg | 0.362 (-0.047–0.771) | 0.914 (0.582–1.000) | 0.756 (0.565–0.947) | 1.984 (1.144–2.823) | Reference |
|  | LGBM | 0.520 (0.142–0.898) | 0.867 (0.623–1.000) | 0.801 (0.625–0.976) | 2.234 (1.387–3.080) | 0.169 (-0.230–0.568) |
|  | MLP | 0.551 (0.142–0.960) | 0.907 (0.670–1.000) | 0.814 (0.649–0.980) | 2.359 (1.430–3.288) | 0.222 (-0.262–0.705) |

| **Table S8.** *(Continued.)* | | | | | | |
| --- | --- | --- | --- | --- | --- | --- |
|  |  | **Sensitivity** | **PPV** | **NPV** | **Diagnostic OR** | **NRI** |
| **Mechanical ventilation** | | |  |  |  |  |
| (-) | LogReg | 0.628 (0.392–0.864) | 0.858 (0.735–0.981) | 0.797 (0.680–0.914) | 2.304 (1.687–2.922) | Reference |
|  | LGBM | 0.470 (0.209–0.730) | 0.835 (0.661–1.000) | 0.734 (0.617–0.850) | 1.941 (1.418–2.464) | -0.140 (-0.366–0.086) |
|  | MLP | 0.593 (0.322–0.864) | 0.839 (0.711–0.966) | 0.782 (0.656–0.909) | 2.203 (1.557–2.848) | -0.037 (-0.206–0.132) |
| (+) | LogReg | 0.355 (0.198–0.512) | 0.554 (0.421–0.687) | 0.819 (0.762–0.876) | 1.893 (1.625–2.161) | Reference |
|  | LGBM | 0.534 (0.381–0.688) | 0.656 (0.566–0.746) | 0.863 (0.808–0.917) | 2.175 (1.897–2.453) | 0.195 (0.028–0.362) |
|  | MLP | 0.428 (0.251–0.605) | 0.589 (0.478–0.700) | 0.836 (0.775–0.897) | 1.987 (1.695–2.279) | 0.074 (-0.068–0.217) |
| **Vasopressor** | |  |  |  |  |  |
| (-) | LogReg | 0.526 (-0.191–1.000) | 0.557 (-0.090–1.000) | 0.964 (0.899–1.000) | 2.673 (2.075–3.270) | Reference |
|  | LGBM | 0.465 (-0.234–1.000) | 0.545 (-0.147–1.000) | 0.959 (0.896–1.000) | 2.674 (2.098–3.250) | -0.033 (-0.719–0.652) |
|  | MLP | 0.748 (0.128–1.000) | 0.678 (0.089–1.000) | 0.981 (0.936–1.000) | 2.863 (2.254–3.472) | 0.138 (-0.276–0.552) |
| (+) | LogReg | 0.405 (0.254–0.556) | 0.661 (0.558–0.764) | 0.777 (0.714–0.840) | 1.848 (1.566–2.129) | Reference |
|  | LGBM | 0.510 (0.396–0.623) | 0.716 (0.641–0.791) | 0.808 (0.754–0.862) | 2.040 (1.808–2.272) | 0.094 (-0.011–0.198) |
|  | MLP | 0.435 (0.303–0.567) | 0.677 (0.597–0.757) | 0.785 (0.721–0.849) | 1.894 (1.640–2.149) | 0.024 (-0.066–0.114) |
| The values are expressed as mean (95% CI).  Abbreviations: FPR, false positive rate; LGBM, light gradient-boosting machine; LogReg, logistic regression; MLP, multi-layer perceptron; NPV, negative predictive value; NRI, net reclassification improvement; OR, odds ratio; PPV, positive predictive value. | | | | | | |

**Supplementary Figures**


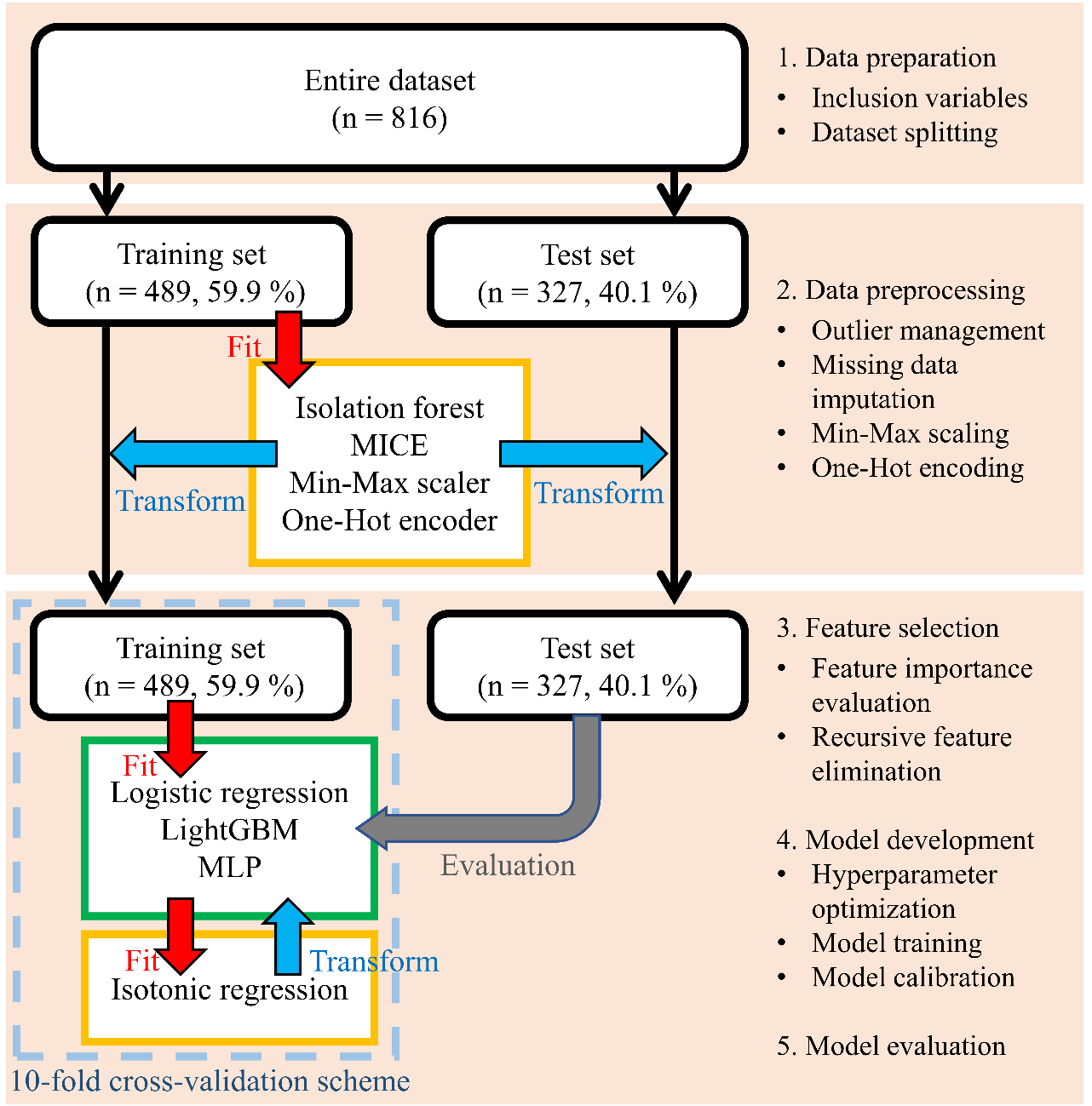


**Figure S1. Workflow diagram including data preprocessing and model development.**

**
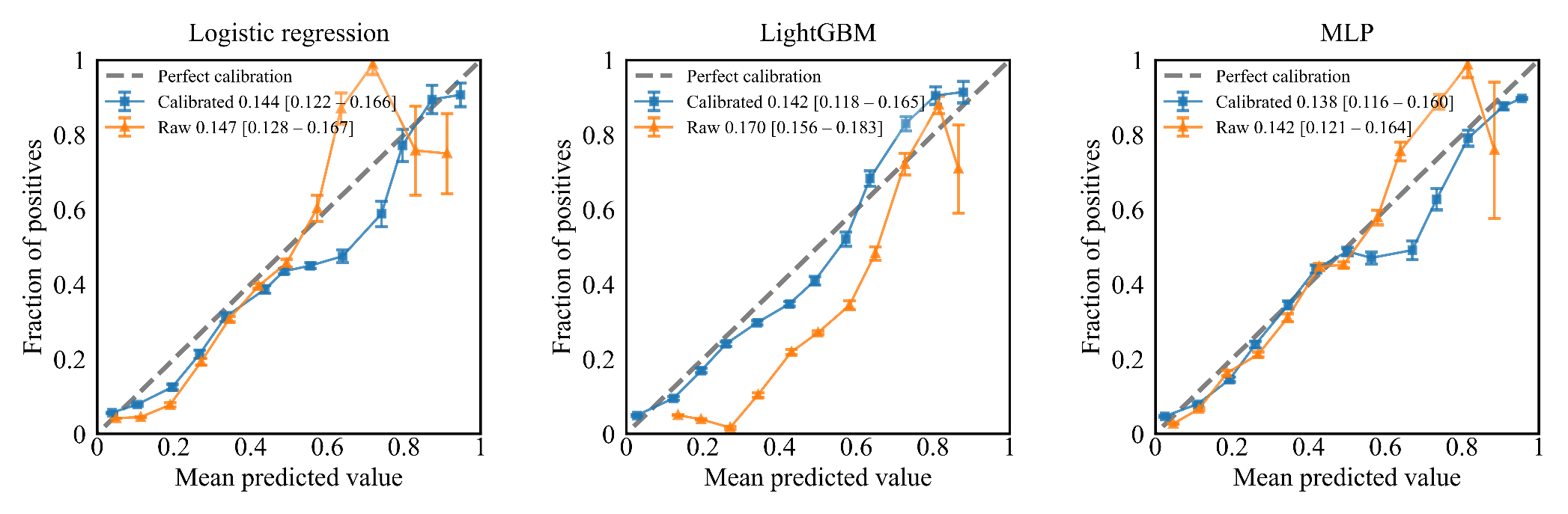
**

**Figure S2. Calibration curves before and after calibration of the models.** Orange and blue lines represent calibration curves before (“Raw”, triangle markers) and after (“Calibrated”, cubic markers) calibration of the models, respectively. Error bars represent 95% confidence interval (CI) of the calibration curves, and the legend displays mean [95% CI] of the Brier scores for model prediction. Abbreviations: LightGBM, light gradient-boosting machine; LogReg, logistic regression; MLP, multi-layer perceptron.

**
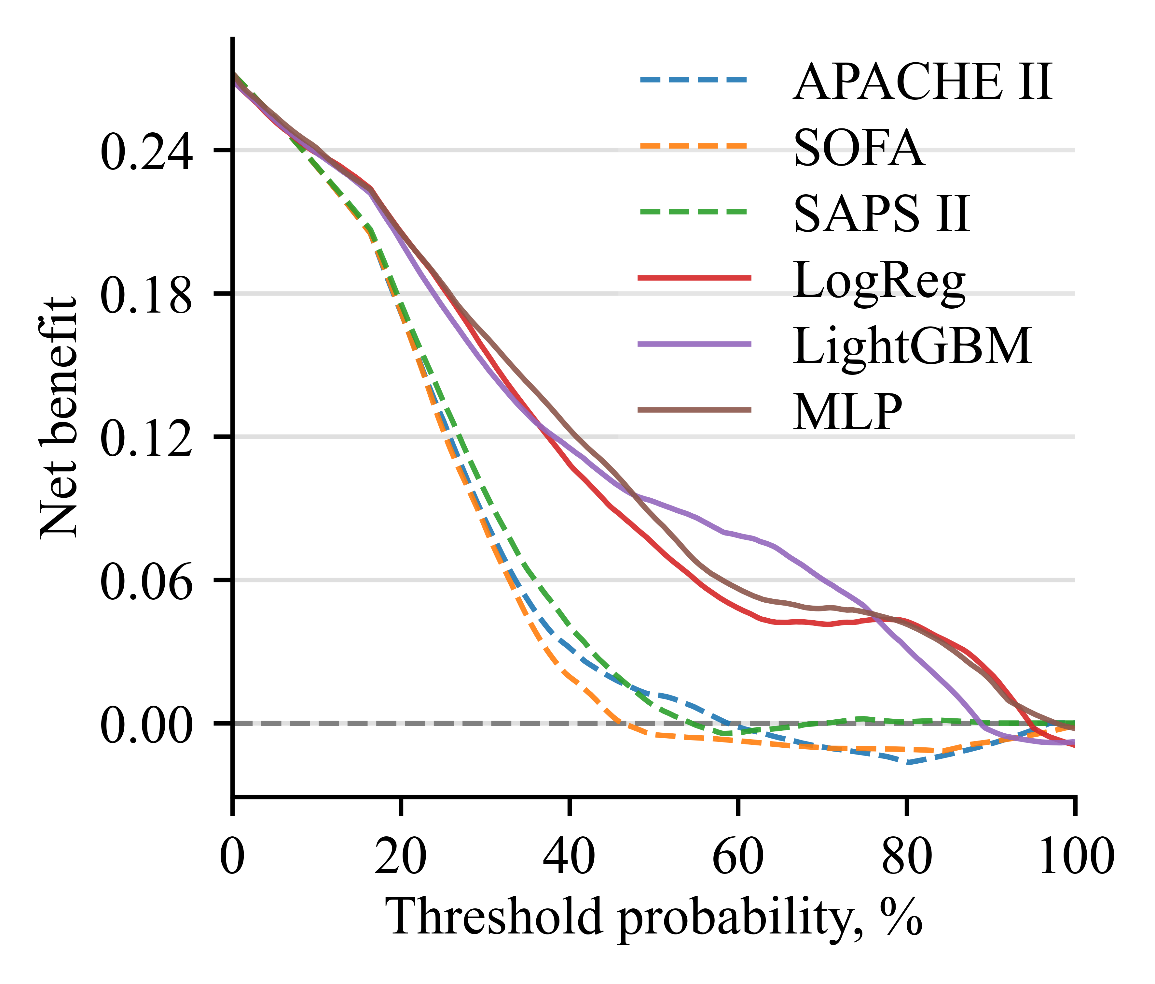
**

**Figure S3. Decision curves for scoring systems and tested models.**

**
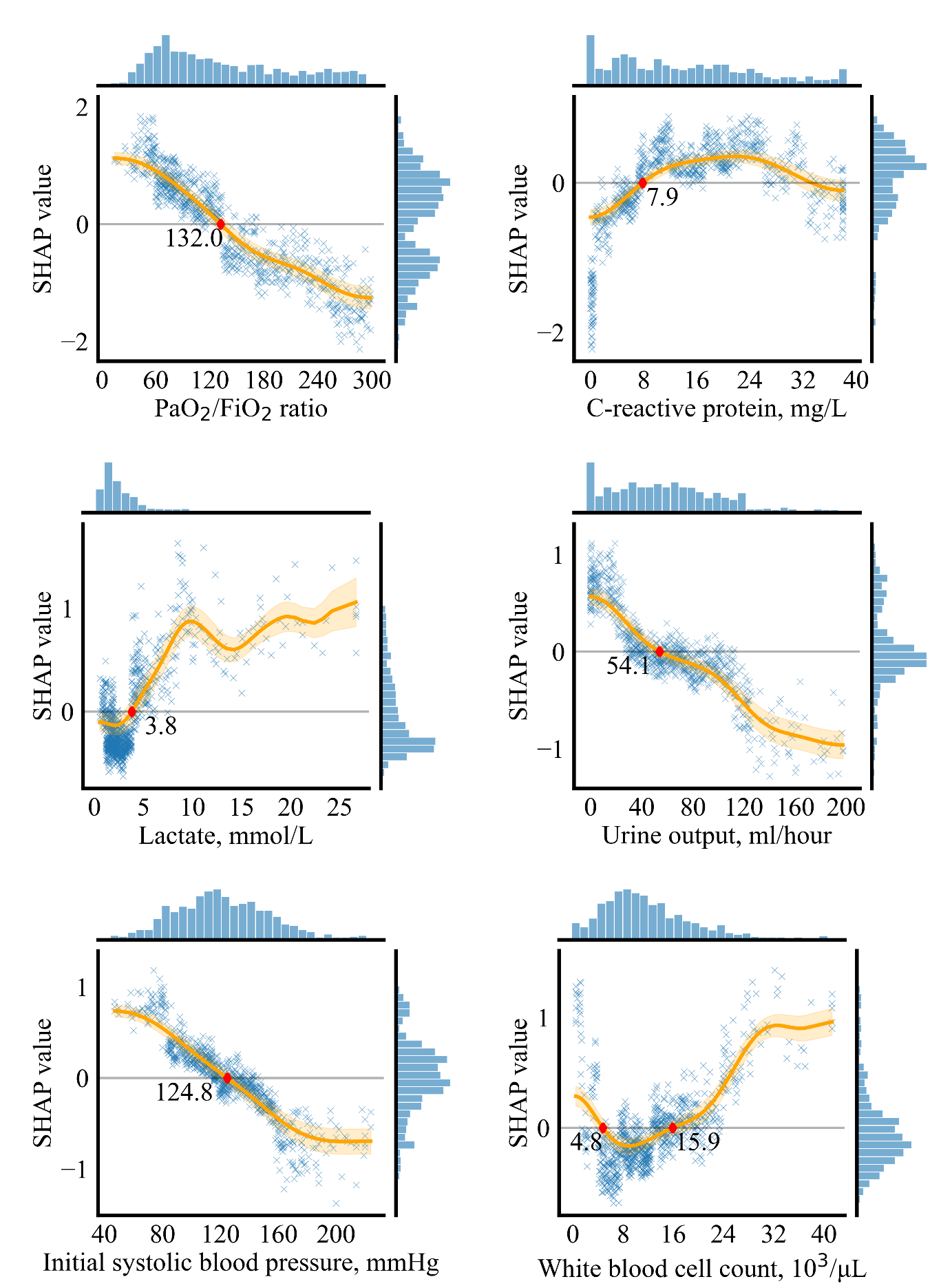
**

**Figure S4. Partial SHAP dependence plots for top-six important variables.** Histograms on the right and top axes of each plot indicate the distributions of the SHAP and variable values, respectively. Scatter plots with regression lines are represented by orange lines of mean and SD shade illustrated for continuous variables.

**
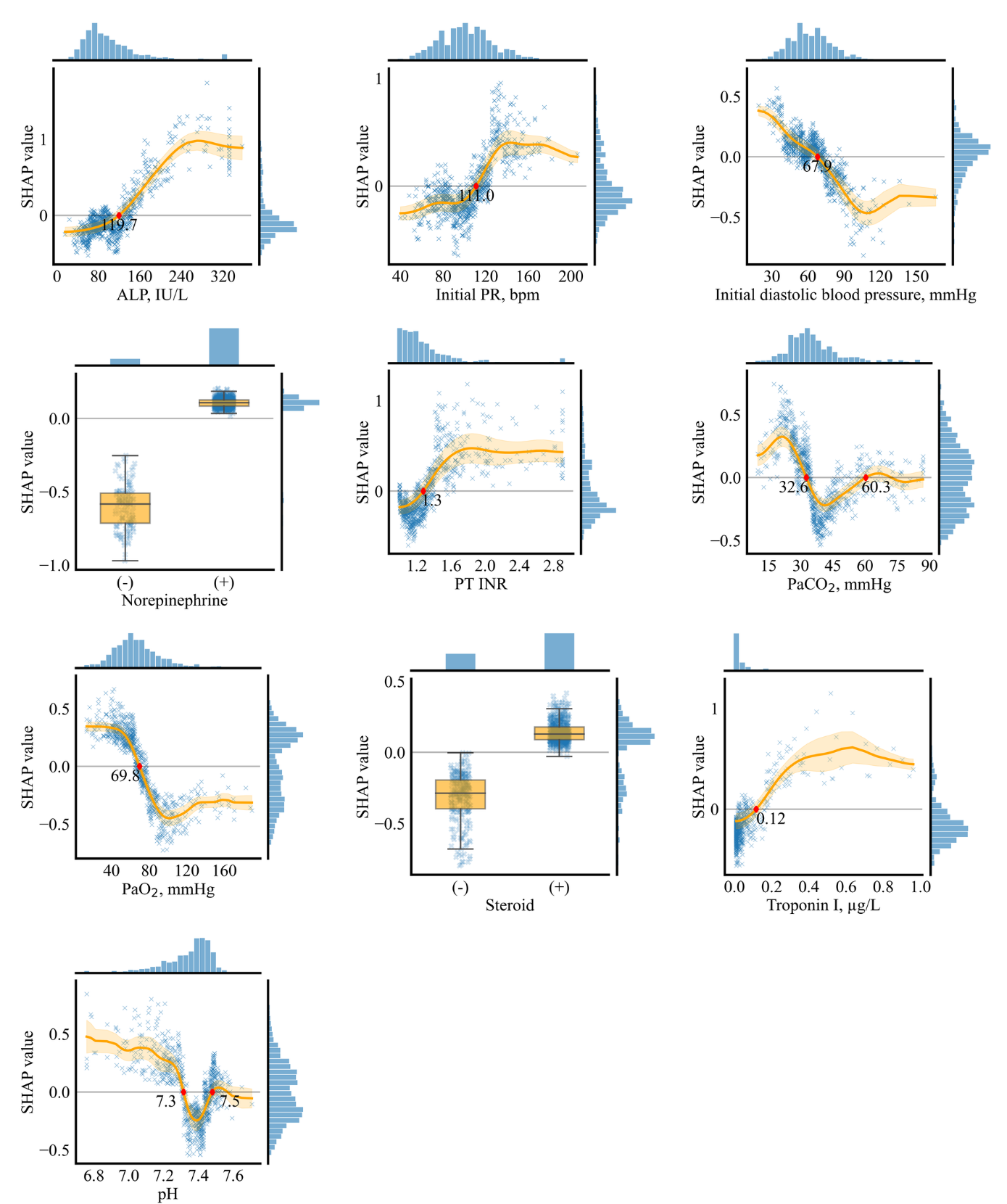
**

**Figure S5. Partial SHAP dependence plots for the selected variables.** Histograms on the right and the top axes of each plot indicate distributions of SHAP and variable values, respectively. For continuous variables, scatter plots with regression lines represented with orange lines of mean and shades of SD are illustrated. Red diamonds represent cut-off values. For categorical variables, scatter plots with box plots with whiskers of 1.5 times the interquartile ranges are illustrated. Abbreviations: ALP, alkaline phosphatase; PR, pulse rate; PT INR, international normalized ratio of prothrombin time.


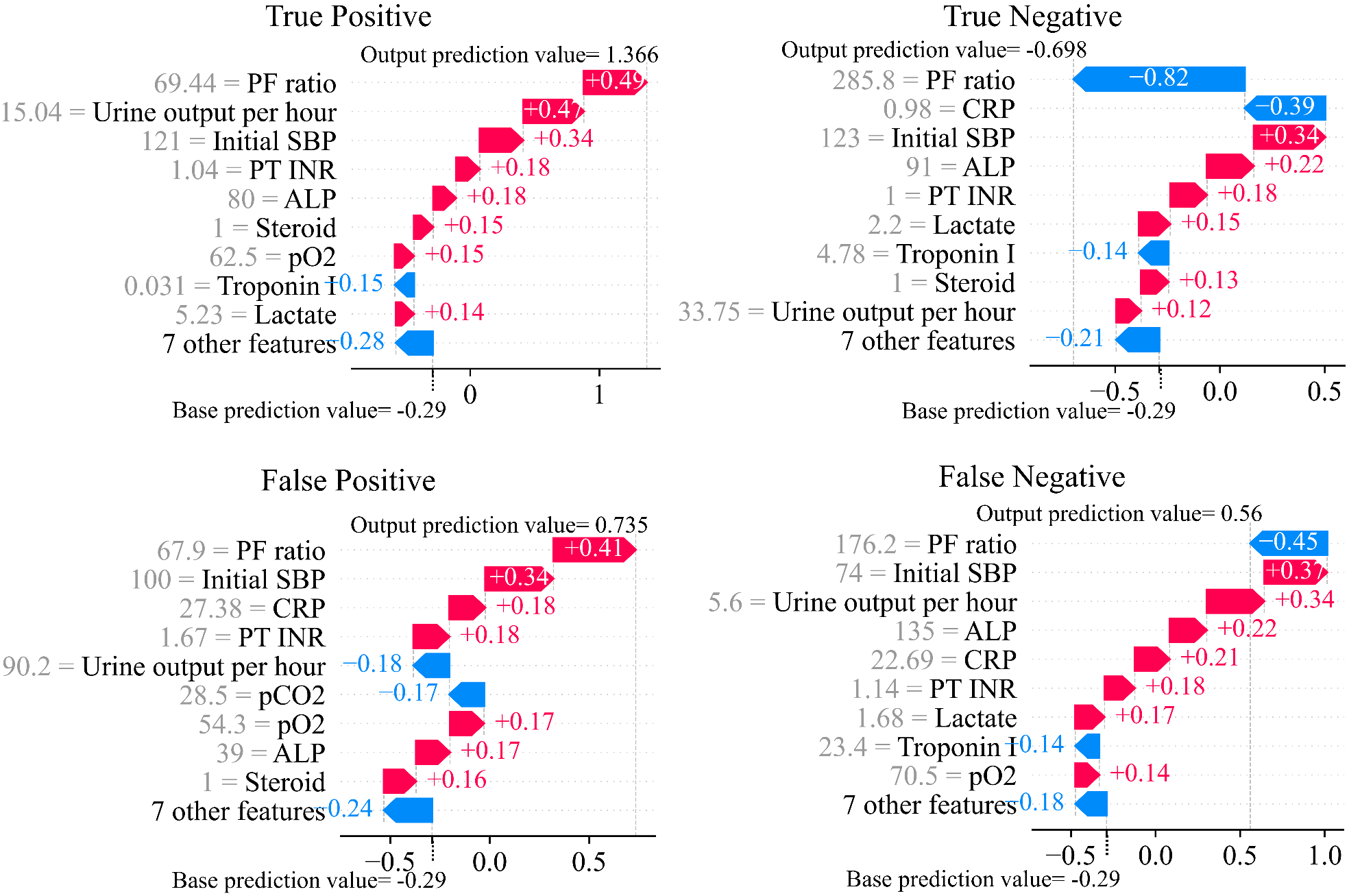


**Figure S6. Local interpretability of the developed gradient-boosted tree-based model.** The cut-off threshold for the model’s decision was set at a false positive rate of 20%.
